# Long noncoding RNA ELDR promotes cell cycle progression in normal oral keratinocytes through induction of a CTCF-FOXM1-AURKA signaling axis

**DOI:** 10.1016/j.jbc.2022.101895

**Published:** 2022-04-01

**Authors:** Subhayan Sur, Robert Steele, Ben C.B. Ko, Jinsong Zhang, Ratna B. Ray

**Affiliations:** 1 Departments of Pathology, Saint Louis University, Missouri, USA; 2 Department of Applied Biology and Chemical Technology, The Hong Kong Polytechnic University, Hong Kong, SAR, PR China; 3 Departments of Pharmacology and Physiology, Saint Louis University, Missouri, USA

**Keywords:** normal oral keratinocytes (NOK), EGFR long non-coding downstream RNA (ELDR), Aurora kinase A (AURKA), Forkhead box M1 (FOXM1), cell cycle, AURKA, Aurora kinase A, CCNB1, cyclin B1, CDC25C, cell division cycle 25C, CDK1, cyclin-dependent kinase 1, CTCF, CCCTC-binding factor, EGFR, epidermal growth factor receptor, ELDR, EGFR long noncoding downstream RNA, FDR, false discovery rate, FOXM1, Forkhead box M1, GO, Gene Ontology, GSEA, gene set enrichment analysis, lncRNA, long noncoding RNA, NOK, normal oral keratinocytes, NOK-ELDR, NOKs stably expressing ELDR, PLK1, Polo-like kinase 1, PID, Pathway Interaction Database, qRT-PCR, quantitative real-time reverse transcription PCR

## Abstract

Long noncoding RNAs (lncRNAs) have gained widespread attention as a new layer of regulation in biological processes during development and disease. The lncRNA ELDR (EGFR long noncoding downstream RNA) was recently shown to be highly expressed in oral cancers as compared to adjacent nontumor tissue, and we previously reported that ELDR may be an oncogene as inhibition of ELDR reduces tumor growth in oral cancer models. Furthermore, overexpression of ELDR induces proliferation and colony formation in normal oral keratinocytes (NOKs). In this study, we examined in further detail how ELDR drives the neoplastic transformation of normal keratinocytes. We performed RNA-seq analysis on NOKs stably expressing ELDR (NOK-ELDR), which revealed that ELDR enhances the expression of cell cycle–related genes. Expression of Aurora kinase A and its downstream targets Polo-like kinase 1, cell division cycle 25C, cyclin-dependent kinase 1, and cyclin B1 (CCNB1) are significantly increased in NOK-ELDR cells, suggesting induction of G2/M progression. We further identified CCCTC-binding factor (CTCF) as a binding partner of ELDR in NOK-ELDR cells. We show that ELDR stabilizes CTCF and increases its expression. Finally, we demonstrate the ELDR-CTCF axis upregulates transcription factor Forkhead box M1, which induces Aurora kinase A expression and downstream G2/M transition. These findings provide mechanistic insights into the role of the lncRNA ELDR as a potential driver of oral cancer during neoplastic transformation of normal keratinocytes.

The whole genome sequencing project has identified more than 98% of human genome that does not encode proteins (1). However, a small number of the noncoding regions have only been functionally annotated resulting in obscurity of the blueprint of life in human genome. Although initially the importance of the noncoding regions were overlooked, recent studies showed about 98% disease-associated genomic variation in the noncoding region (2). Long noncoding RNAs (lncRNAs) are a subclass of the noncoding RNA which are more than 200 nucleotides in length and mainly transcribed by RNA polymerase II (3). The lncRNAs have gained widespread attention now-a-days as a new layer of regulation in biological processes thereby challenging the concept that protein-coding genes are the sole contributors in development and diseases (4, 5, 6, 7). The lncRNA functions as a microRNA sponge to weaken regulations of microRNAs on mRNAs, directly or indirectly interacts with DNAs, RNAs, or proteins thereby regulating cellular homeostasis (4, 5, 6). The lncRNADisease database (www.cuilab.cn/lncrnadisease) enlisted nearly 3000 disease-associated lncRNAs in association with more than 300 human diseases. Most of the lncRNAs possess several functions. The lncRNA ANRIL is involved in cancers, diabetes, and cardiovascular disease, lncRNA H19 in atherosclerosis, coronary artery diseases, regulation in blood pressure, and cancer, lnc-NR2F in developmental disorders, and HOTAIR in cancers (8). Several studies suggested the role of lncRNAs in diagnosis, prognosis and treatment. The lncRNA HOTAIR (NCT03469544) and CCAT1 (NCT04269746) are also in clinical trials for thyroid and colorectal cancer diagnostic biomarkers studies. However, the lncRNA research in oral neoplasia is still in the emerging stage. How lncRNAs maintain normal cellular homeostasis and how deregulation of the lncRNA initiates disease progression are still largely unknown.

We recently reported a potential oncogenic role of ELDR in oral cancer (9). Earlier studies indicated important roles of ELDR in mouse brain development and neural cell differentiation (10, 11, 12). The gene is closely localized at downstream of epidermal growth factor receptor (EGFR) gene in chromosome 7 in the opposite strand. The ELDR is highly expressed in oral cancer samples compared to normal cells and induces cell proliferation by increasing EGFR and ILF3/cyclin E1 signaling (9). We further showed that intratumor delivery of ELDR siRNA regressed oral squamous carcinoma tumor growth in mice. Exogenous expression of ELDR in normal oral keratinocytes (NOKs) induces cell proliferation, although the mechanism remains unknown. In this study, we aimed to examine how ELDR drives toward oncogenesis in NOKs. We observed that overexpression of ELDR in NOK induces Aurora kinase A (AURKA) expression through CCCTC-binding factor (CTCF)/Forkhead box M1 (FOXM1) axis resulting in induction of G2/M progression and increased proliferation. To our knowledge, this is first study indicating an important growth promoting role of the ELDR in normal cells which is a necessary step for neoplastic transformation.

## Results

### ELDR changes global transcriptome profile in NOKs

**Figure 1:**
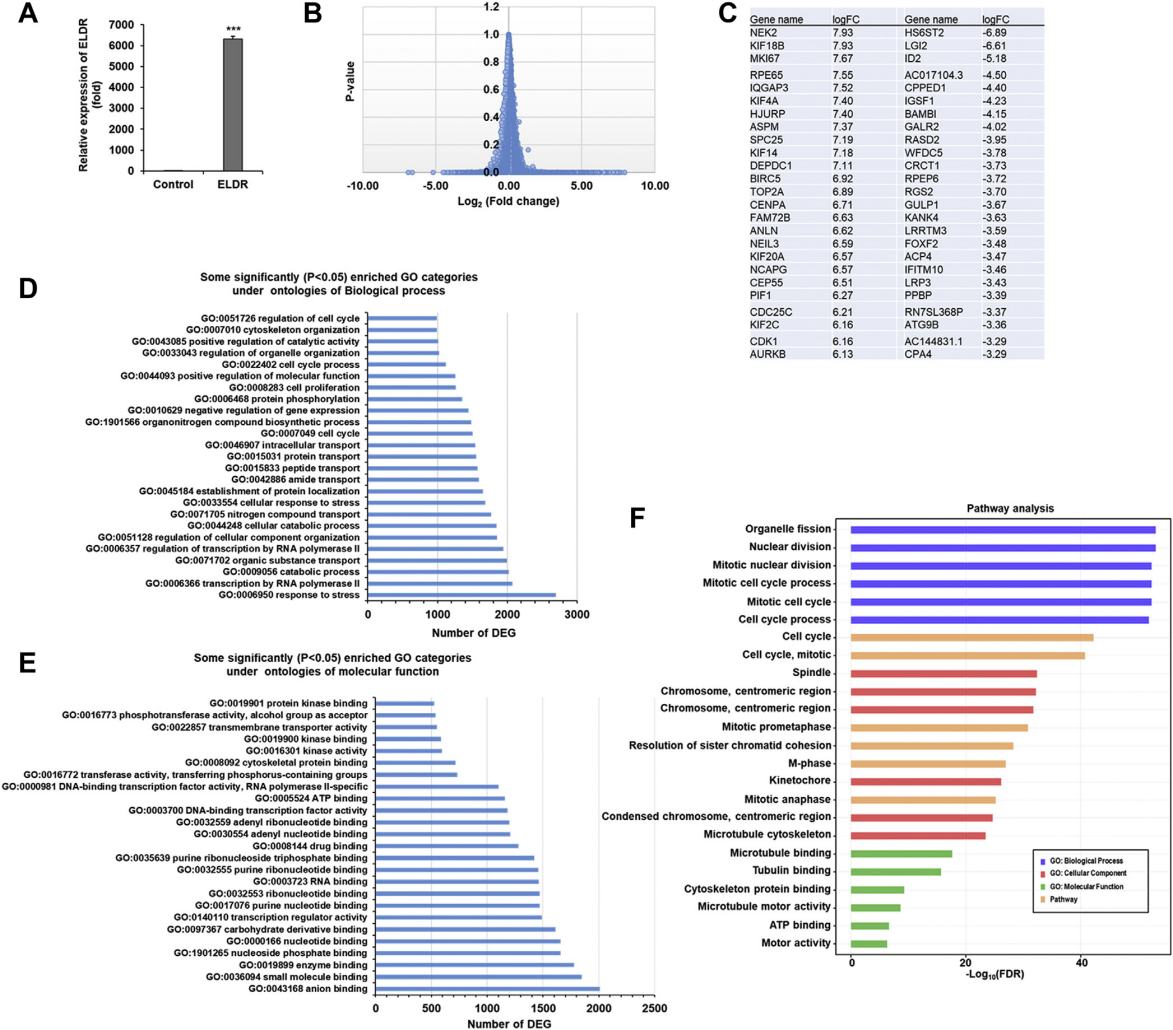
**ELDR changes transcriptome profile in NOK.***A*, ELDR plasmid DNA was stably transfected, treated with G418, and pooled as an established cell line (NOK-ELDR). Relative expression of ELDR in stably overexpressed cells was analyzed by qRT-PCR. Technical triplicates were used, and 18S rRNA was used as an internal control. Small bar indicates standard error (∗∗∗*p* < 0.001). *B*, global changes in transcriptome profile of 15,219 genes in ELDR overexpressed cells (NOK-ELDR) as compared to control NOK. *C*, list of top 25 genes significantly upregulated or downregulated genes following overexpression of ELDR in NOK. *D*, some top significantly enriched Gene Ontology (GO) categories (*p*< 0.05) under ontology of Biological process; *E*, molecular function in ELDR over expressed NOK. *F*, pathway analysis showed cell cycle regulation as most enriched function of genes upregulated in NOK-ELDR cells. The analysis was performed using the ToppGene server. The top six enriched functions under each category were plotted. ELDR, EGFR long noncoding downstream RNA; NOK, normal oral keratinocytes; NOK-ELDR, NOKs stably expressing ELDR.

We generated ELDR stably expressing NOK (NOK-ELDR) (Fig. 1*A*) for next generation RNA sequence analysis. Vector transformed cells was used as a control. We also used two different passage number of stable cells (p6 and p19) in the RNA-seq analysis. The RNA-seq analysis generated a set of 15,219 genes (Fig. 1*B*). Differential expression analysis was performed to analyze for differences between conditions, and the results were filtered for only those genes with Benjamini–Hochberg false discovery rate–adjusted *p*-values less than or equal to 0.05. Out of annotated 15,219 transcripts, 9322 genes were significantly altered in NOK-ELDR cells as compared to the WT cells. Among these, 448 genes are significantly upregulated (log_2_ fold-changes≥ 2, *p* < 0.05), and 137 genes are significantly downregulated (log_2_ fold-changes≤ 2, *p* < 0.05), and we presented top 25 genes (Fig. 1*C*). We noted similar expression from p6 and p19 stable cells.

For each contrast extracted with Limma, global perturbations in known Gene Ontology (GO) terms, and KEGG pathways were detected using the R/Bioconductor package GAGE to test for changes in expression of the reported log_2_ fold-changes reported by Limma in each term *versus* the background log_2_ fold-changes of all genes found outside the respective term. Figure 1, *D* and *E* show significantly enriched (*p* < 0.05) GO terms under ontologies of “Biological process” and “Molecular function”. Among the ontology of “Biological process”, “GO:0006950 response to stress”, “GO:0006366 transcription by RNA polymerase II”, “GO:0009056 catabolic process”, “GO:0071702 organic substance transport”, “GO:0007049 cell cycle”, “GO:0008283 cell proliferation”, and “GO:0051726 regulation of cell cycle” are some top significantly enriched categories in NOK-ELDR (Fig. 1*D*). “GO:0043168 anion binding”, “GO:0036094 small molecule binding”, “GO:0019899 enzyme binding”, “GO:0003723 RNA binding”, “GO:0140110 transcription regulator activity”, “GO:0003700 DNA-binding transcription factor activity”, “GO:0016301 kinase activity”, and “GO:0022857 transmembrane transporter activity” are some top significantly enriched categories under “Molecular function” in NOK-ELDR (Fig. 1*E*).

To independently determine the function of genes that were upregulated in the ELDR-expressing NOK, we performed pathway analysis using the ToppGene server (https://toppgene.cchmc.org/). We analyzed genes that were strongly regulated in the NOK-ELDR with Log2(fold change) > 3 and false discovery rate (FDR) <0.05. As shown in Figure 1*F*, nearly all top-ranked functions and pathways are related to cell cycle regulation. The results also suggest that ELDR target genes play a specific role in regulating the G2/M phase of cell cycle progression.

### ELDR induces G2/M cell cycle in NOKs

**Figure 2:**
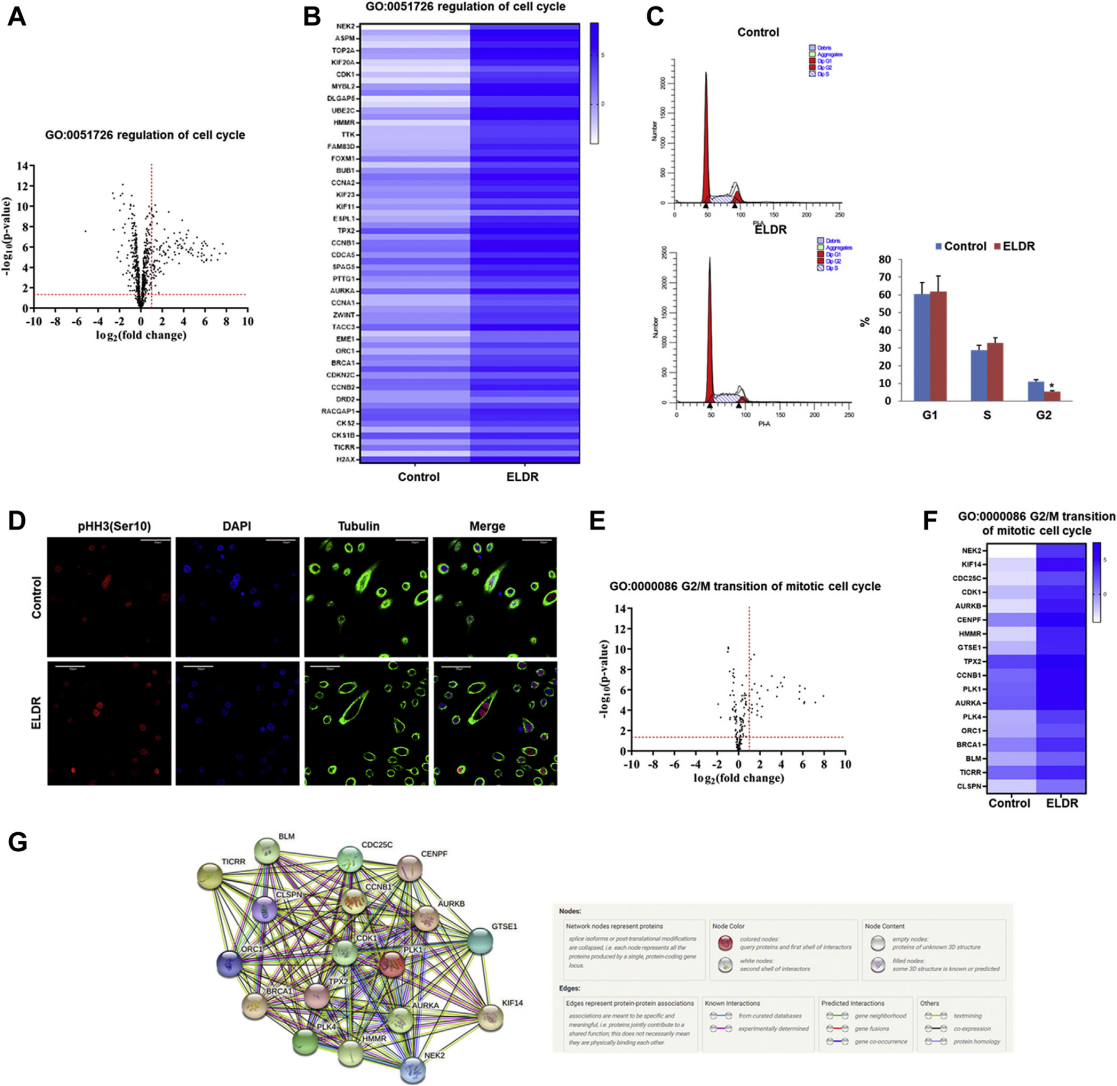
**ELDR overexpression induces G2/M cell cycle in NOK.***A*, volcano plot illustrates significant difference in fold change of genes under “GO:0051726 regulation of cell cycle”. The x-axis represents log_2_ -fold change and y axis is (−log_10_) *p*-value showing statistical significance. Horizontal *dashed red-line* showing *p* =0.05 [−log_10_(0.05) = 1.3] and *vertical dashed red-line* represents fold change at 2 [log_2_(2) = 1]. The absolute 2-fold change and *p*-value 0.05 were considered as the threshold cut-off. *B*, heat map shows expression of some top significantly modulated genes under “GO:0051726 regulation of cell cycle”. *C*, control or ELDR-overexpressed NOK were harvested, fixed, and stained with propidium iodide. DNA content was analyzed by flow cytometry. Results are represented cell population in G1, S, and G2 phases of the cell cycle. Right panel shows percentage of cell population in different phases of cell cycle. Biological duplicates were used, and data are represented as the mean ± SD, *small bar* indicates standard error (∗*p* < 0.5). *D*, control or ELDR-overexpressed NOK were stained with antibody to phospho histone H3 (pHH3) (*red*), tubulin (*green*), and DAPI (*blue*), and representative confocal microscopic images are presented. Magnifications 60×, scale bar 50 μ. *E*, volcano plot illustrates significant difference in fold change of genes under “GO:0000086 G2/M transition of mitotic cell cycle”. The absolute 2-fold change and *p*-value 0.05 were considered as the threshold cut-off. *F*, heat map shows expression of some top significantly modulated genes under “GO:0000086 G2/M transition of mitotic cell cycle”. *G*, interaction network analyzed by String of the top significantly modulated genes under “GO:0000086 G2/M transition of mitotic cell cycle”. ELDR, EGFR long noncoding downstream RNA; NOK, normal oral keratinocytes.

The RNA-seq data showed that 660 genes were significantly modulated (*p*<0.05) under the “GO:0051726 regulation of cell cycle” category in NOK-ELDR cells compared to the WT cells (Fig. 2*A*). Heatmap analysis shows significant upregulation of cell cycle regulatory genes, such as TOP2A, cyclin-dependent kinase 1 (CDK1), CCNA1/2, CCNB1/2, AURKA, AURKB, FOXM1, and H2AX, in NOK-ELDR cells (Fig. 2*B*). Subsequent flowcytometry analysis showed a significant change in G2/M phase of cell cycle in NOK-ELDR as compared to control NOK (Fig. 2*C*). We also observed increased levels of phospho-histone H3 in NOK-ELDR cells indicating increased mitosis (Figs. 2*D* and S1).

Under “GO:0000086 G2/M transition of mitotic cell cycle”, RNA-seq data showed significant modulation of 121 genes in NOK-ELDR cells (Fig. 2*E*). Heatmap analysis showed significant alteration of genes, including cell division cycle 25C (CDC25C), CDK1, AURKA, AURKB, cyclin B1 (CCNB1), and Polo-like kinase 1 (PLK1), in NOK-ELDR cells as compared to control cells (Fig. 2*F*). String analysis showed extensive network association of these genes under “G2/M transition of mitotic cell cycle”, suggesting their functional importance for this biological process (Fig. 2*G*).

**Figure 3:**
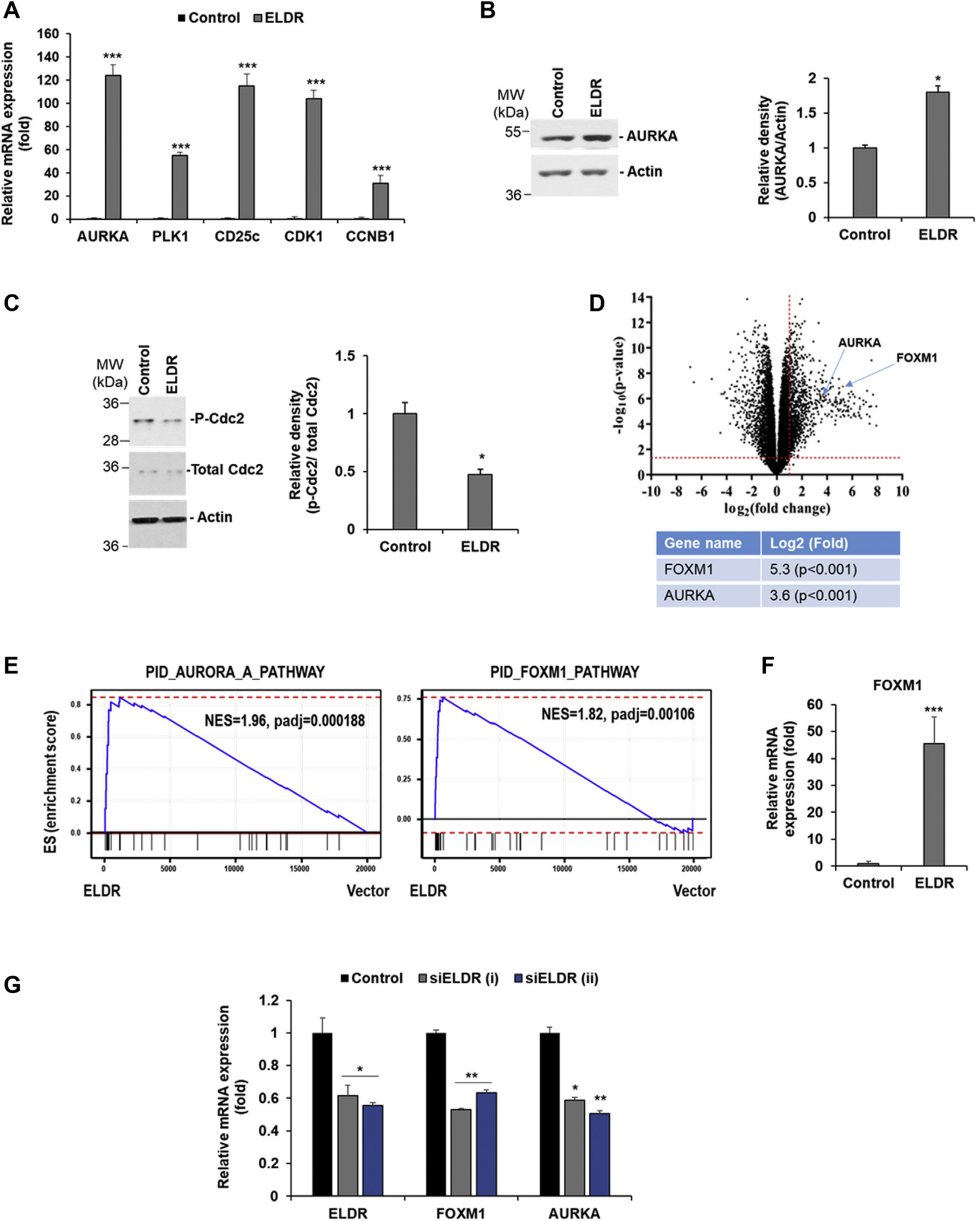
**Overexpression of ELDR induces FOXM1-AURKA signaling.***A*, relative mRNA expression of AURKA, PLK1, CDC25C, CDK1, and CCNB1 in ELDR-overexpressed NOK was analyzed by qRT-PCR as compared to NOK. 18S rRNA was used as an internal control. *Small bar* indicates standard error (∗∗∗*p* < 0.001). Experiments were repeated three times with technical triplicates. *B*, control or ELDR-overexpressed NOK lysates were subjected to Western blot analysis for AURKA and (*C*) phospho-CDK1 and total CDK1 (Cdc2) using specific antibodies. The membranes were reprobed with actin as an internal control. Right panel shows quantitation. *Small bar* indicates standard error (∗*p* < 0.05). Experiments were repeated two times. *D*, the expression of AURKA and FOXM1 was shown (*red dots*) among a total 15,219 genes in the Volcano plot generated from the RNA seq data of NOK-ELDR *versus* NOK. *Horizontal and vertical dashed red lines* represent absolute 2-fold change and *p*-value 0.05 as the threshold cut-off. *E*, AURKA and FOXM1 pathway genes are strongly upregulated in ELDR-expressing NOK cells. Gene set enrichment analysis was performed using the “fgsea” R package with genes ranked based on their differential expression in NOK-ELDR and control NOK cells against the Pathway Interaction Database (PID). The AURORA A (AURKA) and FOXM1 pathways were among the upregulated pathways in cells expressing ELDR. *F*, relative mRNA expression of FOXM1 in ELDR-overexpressed NOK analyzed by qRT-PCR as compared to NOK. 18S rRNA was used as an internal control. *Small bar* indicates standard error (∗∗∗*p* < 0.001). Experiments were repeated three times with technical triplicates. *G*, NOK-ELDR cells were transfected with two different siRNAs to ELDR and after 48 h mRNA expression of ELDR, FOXM1, and AURKA were analyzed by qRT-PCR. 18S rRNA was used as an internal control. Relative gene expression is shown with mean value ±standard error (∗*p* < 0.05; ∗∗*p* < 0.01). Experiments were repeated three times with technical triplicates. AURKA, Aurora kinase A; CCNB1, cyclin B1; CDC25C, cell division cycle 25C; CDK1, cyclin-dependent kinase 1; EGFR, epidermal growth factor receptor; ELDR, EGFR long noncoding downstream RNA; FOXM1, Forkhead box M1; NOK, normal oral keratinocytes; NOK-ELDR, NOKs stably expressing ELDR; PLK1, Polo-like kinase 1; qRT-PCR, quantitative real-time reverse transcription PCR.

We validated expression of key regulatory genes involved in G2/M-phase cell cycle. We observed a significant enhancement of AURKA, PLK1, CDC25C, CDK1, and CCNB1 mRNA expression in NOK-ELDR cells (Fig. 3*A*). The serine-threonine kinase, AURKA, is one of the important mitosis regulators and is upregulated in many cancers including oral cancer (13, 14). In the signaling cascade, AURKA is the most upstream regulator. It activates PLK1 by phosphorylation, which regulates sequential phosphorylation and dephosphorylation events resulting in activation of the effector molecule CDK1/Cdc2 by dephosphorylation (13). We observed significant upregulation of AURKA protein and reduction of the inhibitory phosphorylation of Cdc2 in NOK-ELDR cells, suggesting induction of G2/M phase of cell cycle (Fig. 3, *B* and *C*).

We next examined how ELDR regulates G2/M phase genes. RNA-seq data showed significant overexpression of FOXM1 gene in the NOK-ELDR cells (Fig. 3*D*). We performed gene set enrichment analysis (GSEA) to further test the hypothesis that ELDR regulates cell cycle by regulating AURKA and FOXM1. The GSEA analysis was performed using NOK-ELDR and control cells. We focused on gene pathways contained in the Pathway Interaction Database (PID) (15). Consistent with the hypothesis, both AURKA-regulated (normalized enrichment score=1.96, *p*=0.000188) and FOXM1-regulated (normalized enrichment score=1.82, *p*=0.00106) pathway genes were strongly upregulated in NOK cells expressing ELDR (Fig. 3*E*). FOXM1 is a transcription factor that transactivates AURKA and other G2/M genes like PLK1, CDK1, and CCNB1 (16, 17, 18). High FOXM1 expression was reported in many cancers including oral cancer (19, 20). We validated the FOXM1 expression by quantitative real-time reverse transcription PCR (qRT-PCR). A significant overexpression of FOXM1 was evident in NOK-ELDR cells (Fig. 3*F*). Further, knocked down of ELDR in NOK-ELDR cells resulted in significant reduction of FOXM1 and AURKA transcription (Fig. 3*G*). Together, our results suggest that ELDR induces FOXM1 expression which might subsequently induce AURKA expression for progression of cell cycle through G2/M phase.

### ELDR enhances CTCF expression

**Figure 4:**
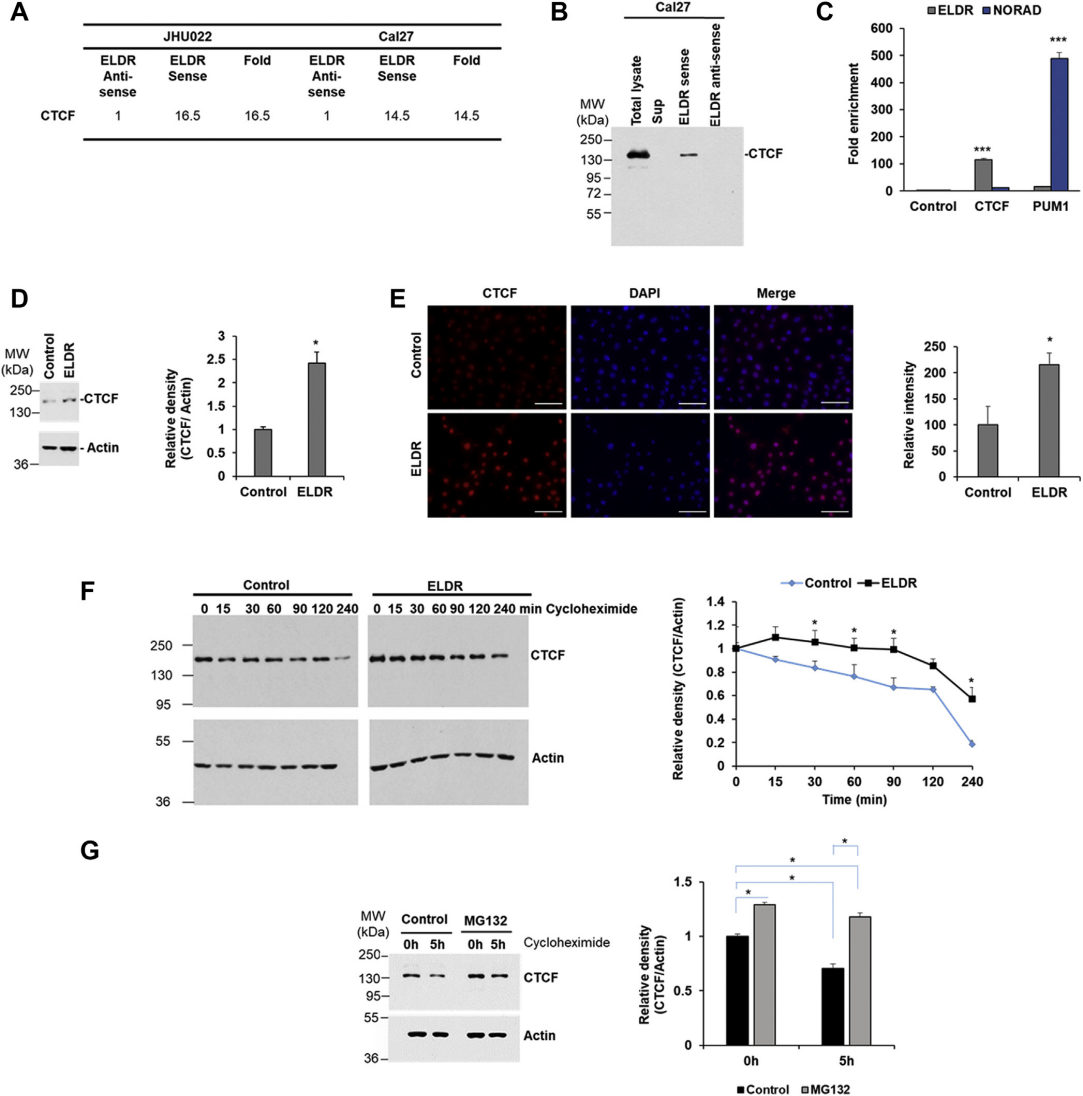
**ELDR regulates CTCF expression by physical interaction.***A*, spectrum count of CTCF protein analyzed by liquid chromatography mass spectrometry from ELDR sense *versus* antisense RNA pulled down lysates of oral cancer cells JHU022 and Cal27. *B*, cell lysates from Cal27 cells were incubated with biotinylated ELDR sense and antisense RNA, pulled down, and subjected to Western blot analysis for the CTCF using specific antibody. Experiments were repeated two times. *C*, NOK-ELDR lysate was immunoprecipitated against control (IgG-isotype control), CTCF, or PUM1 antibodies, and RNA was isolated from the precipitates. Relative expressions of ELDR and NORAD were analyzed by qRT-PCR. Experiments were repeated two times with technical triplicates. Small bar indicates standard error (∗∗∗*p* < 0.001). *D*, control or ELDR-overexpressed NOK lysates were subjected to Western blot analysis for CTCF using specific antibody. The membrane was reprobed with actin as an internal control. Right panel shows quantitation*. Small bar* indicates standard error (∗*p* < 0.05). Experiments were repeated two times with technical replicates. *E*, control or ELDR-overexpressed NOK were stained with antibody to CTCF (*red*) and DAPI (*blue*), and representative fluorescence microscopic images are presented. Magnifications 40×. Scale bar 50 μm. Right panel shows quantitation. *Small bar* indicates standard error (∗*p* < 0.05). *F*, control or ELDR-overexpressed NOK were treated with cycloheximide and harvested at indicated time points. Cell lysates were subjected to Western blot analysis for CTCF using specific antibody. The membrane was reprobed with actin as an internal control. Right panel shows quantitation. Small bar indicates standard error (∗*p* < 0.05). Experiments were repeated two times with technical duplicates. *G*, lysates from NOK with or without MG132 (10 μM) along with cycloheximide chase were analyzed by Western blot using CTCF at indicated time points. The membrane was reprobed with actin as an internal control. Right panel shows quantitation. Small bar indicates standard error (∗ *p* < 0.05). Experiments were repeated two times. ELDR, EGFR long noncoding downstream RNA; NOK, normal oral keratinocytes; NOK-ELDR, NOKs stably expressing ELDR; CTCF, CCCTC-binding factor.

We next examined how ELDR regulates FOXM1 expression. FOXM1 promoter is transcriptionally activated by CTCF in hepatocellular carcinoma (21). In our proteomics analysis by LC-MS, we found CTCF binds with only the sense strand of ELDR (Figs. 4*A* and S2). The zinc-finger protein CTCF has both RNA and DNA binding ability and regulates transcription of many genes (22, 23). We verified the interaction by RNA pull-down assay followed by Western blot analysis. We observed an abundance of CTCF in RNA protein complex in Cal27 cells (Fig. 4*B*). Furthermore, RNA immunoprecipitation with antibodies against CTCF showed significant enrichment of ELDR RNA in the immunoprecipitates of NOK-ELDR cells (Fig. 4*C*). An unrelated lncRNA NORAD in the precipitates was used as a control, and no interaction between CTCF and NORAD was observed. We used PUM1, a known binding partner of NORAD (24), and we observed enrichment of NORAD, not ELDR, in the immunoprecipitates of NOK-ELDR cells, further confirming the specificity (Fig. 4*C*). *In-silico* analysis (s.tartaglialab.com/) predicted potential interaction between ELDR exon-2 and C-terminal KRRGRP-type AT-hook (626–677) of CTCF. We generated C′-terminal deleted construct of CTCF (Δ CTCF) of 571 amino acids and cloned into pCAGGS- 3 × FLAG plasmid (Fig. S3*A*). The NOK-ELDR cells were transfected with full length (Fl-CTCF-Flag) or ΔCTCF-Flag. The RNA pull-down assay was performed followed by Western blot analysis with Flag antibody. Our data suggested an interaction of ELDR with Fl-CTCF but not with ΔCTCF (Fig. S3*B*). Together these results suggest a specific interaction between ELDR and CTCF.

Next, we examined the consequence of ELDR and CTCF interaction in NOK. We did not observe an alteration of CTCF mRNA expression following ELDR overexpression either in RNA seq analysis or qRT-PCR (Fig. S4, *A* and *B*). However, a significant overexpression of CTCF protein expression was noted in NOK-ELDR cells as compared to control cells (Fig. 4*D*). Immunostaining further verified increased expression of CTCF mostly in nucleus of ELDR-overexpressed NOK (Fig. 4*E*). We performed RNA immunoprecipitation assay in cytoplasmic and nuclear fractions of NOK-ELDR cells for ELDR-CTCF interaction and observed a significant enrichment of ELDR RNA in the nuclear immunoprecipitates (Fig. S5). Next, CTCF protein stability assay was performed after blocking endogenous translation by cycloheximide chase at different times. We observed that ELDR enhances the stability of CTCF in NOK (Fig. 4*F*). We also examined whether CTCF is degraded by the proteasome. For this, NOK was treated with vehicle (dimethyl sulfoxide) or MG132 proteasome inhibitor. Our result showed the relief of CTCF inhibition following MG132 treatment (Fig. 4*G*) These results suggest that proteasomal degradation of CTCF was impaired in NOK-ELDR, although further work is necessary to elucidate the mechanism.

### ELDR-CTCF axis regulates FOXM1 and AURKA expression in NOK

**Figure 5:**
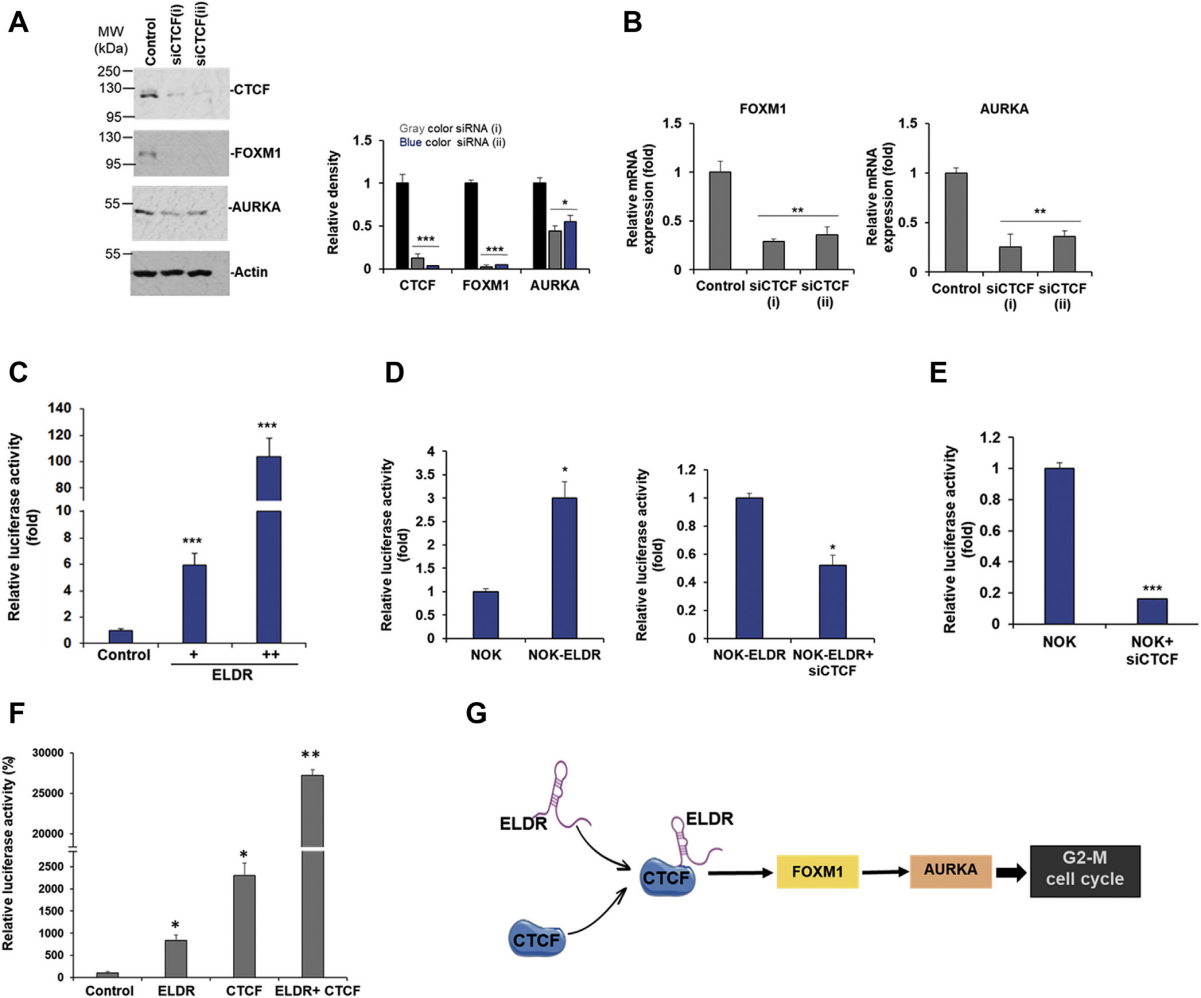
**ELDR-CTCF axis regulates FOXM1 and AURKA expression in NOK.** NOK-ELDR cells were transfected with either control or two different siRNAs to CTCF, and after 48 h, cells were harvested for protein and mRNA analysis. *A*, Western blot analysis for CTCF, FOXM1, and AURKA using specific antibodies. The membrane was reprobed with actin as an internal control. Right panel shows quantitation. *Small bar* indicates standard error (∗*p* < 0.05; ∗∗∗*p* < 0.001). Experiments were repeated two times. *B*, relative mRNA expression of FOXM1 and AURKA in control or CTCF-depleted NOK-ELDR analyzed by qRT-PCR. 18S rRNA was used as an internal control. *Small bar* indicates standard error (∗∗*p* < 0.01). Experiments were repeated three times with technical triplicates. *C*, 293T cells were cotransfected with vector or ELDR plasmid DNA and FOXM1 promoter-luciferase reporter plasmids. After 48 h, relative luciferase activity was measured. Experiments were repeated two times with technical triplicates. *D*, NOK and NOK-ELDR cells were transfected with FOXM1 promoter–luciferase reporter plasmid with or without control or siRNA(i) to CTCF, and after 48 h, relative luciferase activity was measured. Experiments were repeated three times with technical duplicates. *E*, NOK cells were cotransfected with FOXM1 promoter–luciferase reporter plasmids and control or siCTCF(i), and after 48 h, relative luciferase activity was measured. Experiments were repeated three times with technical triplicates. *Small bar* indicates standard error (∗*p* < 0.05; ∗*p* < 0.01, ∗∗∗*p* < 0.001). *F*, 293T cells were cotransfected with FOXM1 promoter–luciferase reporter plasmid DNA, vector, ELDR, CTCF or both ELDR, and CTCF plasmid DNAs. Luciferase activity was measured 48 h posttransfection. Small bar indicates standard error (∗*p* < 0.05; ∗∗*p* < 0.01). Experiments were repeated two times with replicates. *G*, schematic representation shows ELDR and CTCF interaction in NOK resulting in FOXM1–AURKA axis–mediated progression of G2/M cell cycle. CTCF, CCCTC-binding factor; AURKA, Aurora kinase A; CCNB1, cyclin B1; CDC25C, cell division cycle 25C; EGFR, epidermal growth factor receptor; ELDR, EGFR long noncoding downstream RNA; FOXM1, Forkhead box M1; NOK, normal oral keratinocytes; NOK-ELDR, NOKs stably expressing ELDR; qRT-PCR, quantitative real-time reverse transcription PCR.

We further examined the CTCF-FOXM1-AURKA axis in NOK. For this, the CTCF was depleted by two different siRNAs, and a significant knockdown was noted (Figs. 5*A* and S6). We also observed a significant reduction in FOXM1 and AURKA protein expression upon knockdown of CTCF in NOK (Fig. 5*A*). A significant reduction in mRNA expressions of FOXM1 and AURKA were observed in CTCF depleted NOK (Fig. 5*B*). Further, CTCF over expression significantly induced FOXM1 and AURKA mRNA expression in NOK (Fig. S7). To confirm the transcriptional regulation of FOXM1 by ELDR-CTCF axis, FOXM1 promoter region cloned in luciferase reporter plasmid was cotransfected with control or ELDR plasmid DNA in 293T cells. Luciferase activity was higher following overexpression of ELDR in dose-dependent manner, suggesting ELDR-mediated upregulation of FOXM1 promoter activity (Fig. 5*C*). We further confirmed the effect of ELDR in NOK or NOK-ELDR cells. A significant induction in FOXM1 promoter activity was observed in NOK-ELDR cells as compared to NOK, which can be reversed by depleting CTCF (Fig. 5*D*). Depletion of CTCF significantly reduced FOXM1 promoter activity in NOK, as expected (Fig. 5*E*). We further tested whether overexpression of ELDR and CTCF displays additive effect on FOXM1 promoter activity. As shown in Figure. 5*F*, co-expression ELDR and CTCF enhance luciferase activity, suggesting enhancement of FOXM1 expression in presence of both genes. Thus, our results suggested that FOXM1 upregulation and resulting induction of AURKA is mediated through ELDR-CTCF axis (Fig. 5*G*).

## Discussion

The lncRNA ELDR has potential role in oral cancer growth. In this report, we used a NOKs model to explore the mechanism of ELDR in oncogenic transformation. The ELDR increased proliferation by accelerating G2/M phase of cell cycle progression. Mechanistically, ELDR stabilizes CTCF by physical interaction resulting in FOXM1-AURKA–mediated G2/M progression in NOK. The NOKs show a restricted life span in cell culture which is suggested to act as a barrier against a tumorigenic transformation of a normal cell (25). Stable expression of the ELDR in NOK induces its life span in culture. We observed significant change in G2/M phase of cell cycle and increased phosphorylation of histone H3 in NOK-ELDR cells.

The MAGEA4, belong to Class I cancer testis antigens, is upregulated in head and neck cancer. Overexpression of MAGEA4 in NOK stimulates growth by inhibiting cell cycle arrest and apoptosis (26). The squamous epithelia are present in a variety of mammalian locations including oral cavity, larynx, pharynx, and esophagus and regulate development and differentiation. Upon oncogenic stimuli, the keratinocytes need to slip beyond G2/M to initiate differentiation (27). For example, upon exposure to cigarette smoke, a significant shortening of G2/M phase was reported in SCC-15 and SCC-25 cells (28). Cigarette smoke–associated acrolein could induce proliferation and tumorigenic transformation in NOK by increasing EGFR signaling (29). The important step of tumor initiation and progression is transformation of normal cells which begins by proliferating abnormally. In normal cellular homeostasis, hundreds of genes intricately regulate the process of cell division and proliferation by balancing the genes that promote cell proliferation and those that suppress it. During tumorigenic progression, normal cells acquire multiple genetic changes of cancer-associated ‘Hallmarks’ that induce cell division and proliferation (30).

Our RNA-seq analysis revealed a significant modulation of 9322 genes upon ELDR overexpression. GO and pathway analyses showed that most of the modulated genes are associated with cell cycle. We identified significantly modulated 121 genes which are specifically associated with G2/M phase of cell cycle. The AURKA and its downstream signaling genes of the G2/M phase including PLK1, CDC25C, CDK1, and CCNB1 are mostly upregulated in NOK-ELDR cells. The AURKA is a type of serine-threonine kinase which phosphorylates PLK1 leading to a sequential activation of CDC25C, CDK1, and CCNB1 of G2 to M phase transition. Upregulation of AURKA is reported in many cancers including oral cancer as compared to respective normal tissues (13, 14, 31). Enhanced AURKA results in centrosome maturation, mitotic entry, formation and function of the bipolar spindle, cytokinesis, aneuploidy, supernumerary centrosomes, and resistance to apoptosis. AURKA level tightly regulates timing of mitotic entry in embryonic and somatic cells (32). Overexpression of AURKA in G2-arrested *Xenopus* oocytes or mouse oocytes accelerates M-phase (32). Ectopic expression of AURKA induces oncogenic transformation in mouse and rat fibroblasts and is suggested to be a potential therapeutic target against different cancers (33). The transcription factor FOXM1 transactivates AURKA and its downstream signaling genes PLK1, CDK1, and CCNB1 (16, 17, 18). The FOXM1 is required for normal cellular proliferation; however, overexpression of this gene has been implicated as a major predictor of adverse outcomes in 18,000 cancer cases across 39 human malignancies (34, 35). The FOXM1 is responsible for oncogenic transformation and in tumor initiation, progression, metastasis, and therapy resistance (36). Overexpression of FOXM1 in NOK suppressed the tumor suppressor gene p16 (INK4A) (CDKN2A) through promoter hypermethylation (37). It seems that the ELDR may play an important role in upregulation of FOXM1 in NOK. ELDR expression was very low (almost undetectable) in NOK; therefore, we could not perform the ELDR depletion experiment in NOK; however, depletion of ELDR in NOK-ELDR could impair FOXM1 and AURKA expression. We have also shown that ELDR induces G1/S phase by increasing cyclin E1 expression in oral cancer cells (9). Generally, tumor promoting genes that function to drive the cell cycle forward, typically causing cells to proceed from one of the G phases to either chromosome replication (S phase) or chromosome segregation (mitosis) (38). This may depend on normal or cancer context. For example, proteasome inhibitor MG132 induces G1/S arrest in normal mammary cell line MCF10A, while G2/M arrest in breast cancer cells MCF7 (39). It is plausible that ELDR differentially regulates cell cycle in NOKs and oral cancer cells.

We found that the ELDR physically interacts with CTCF and increases CTCF expression by stabilization. The CTCF is an important regulator of chromatin organization and controls gene expression by stabilizing enhancer–promoter interaction (40). The CTCF is essential for embryonic development, and depletion of CTCF gene inhibits normal cell function and organ specific failure in oocytes, lymphocytes, neurons, and cardiomyocytes (41, 42). Overexpression of CTCF was reported to initiate multiple cancer types including breast cancer, hepatocellular carcinoma, lung cancer, prostate cancer, and colorectal cancer (43). The CTCF is degraded by E3 ubiquitin-protein ligase HUWE1 (ARF-BP1) (44). Besides, there are multiple ubiquitination and phosphorylation sites in CTCF that regulate stability and functions [PhosphoSitePlus (PSP)]. We observed the enhancement in CTCF expression in protein level, not in mRNA level. The FOXM1 promoter contains CTCF binding site, and CTCF transcriptionally activates FOXM1 in cancer (21, 43). We observed increased FOXM1 mRNA expression in ELDR overexpressed NOK which was reversed upon CTCF depletion.

Neoplastic transformation and development of cancer from a normal cell is multistep process characterized by progressive series of alterations and then selection for cells with progressively increased capacity for proliferation. Recent studies suggested potential roles of lncRNAs in development and diseases including cancers. Thus, understanding role of lncRNA in normal cells would provide important mechanistic information in understanding and management of the disease. We implanted parental NOK or NOK-ELDR cells in NSG mice. Control cells did not display any tumor, as expected. Mice having NOK-ELDR displayed pea size tumor which did not grow further, suggesting second hit may be required and needs further evaluation. However, lncRNA research is still in the developing stage, and role of the lncRNA in modulation of normal function is not clear. We described here that ELDR as a mediator of a novel proliferation network in the context of normal oral cells when the lncRNA is solely overexpressed. This indicates that ELDR may be one of the potential drivers in oral cancer which is responsible for tumorigenic transformation. However, there are several questions remaining. It will be important to know how ELDR is upregulated in oral cancer and probably in other cancers. In conclusion, tumor promoting lncRNA ELDR has potential role in induction of proliferation in NOKs by increasing G2/M transition of cell cycle when the ELDR is overexpressed. Mechanistically, the ELDR interacts with and stabilizes CTCF resulting in CTCF target gene FOXM1-mediated upregulation of AURKA signaling.

## Experimental procedures

### Cell culture and transfection

NOK cells (kindly gifted by Karl Mugner) were maintained in keratinocyte serum free medium supplemented with epidermal growth factor and bovine pituitary extract (Gibco, Life technologies) and 1% penicillin/streptomycin. Oral cancer cell line Cal27 was purchased from the American Type Culture Collection (ATCC). JHU022 cell line was procured from the Johns Hopkins University. The Cal27 and JHU022 cells were maintained in Dulbecco’s Modified Eagle Medium with 10% FBS and 1% penicillin/streptomycin (Sigma-Aldrich) in a humidified CO_2_ incubator. The 293T cells were purchased from Clontech (Takara Bio) and maintained in Dulbecco’s Modified Eagle Medium supplemented with 10% FBS and 1% penicillin/streptomycin. The cell lines are routinely tested in our laboratory to rule out *mycoplasma* contamination using commercial LonzaMycoAlert *Mycoplasma* Detection kit. ELDR [NR_110,426.1] pcDNA3.1 plasmid DNA or control plasmid DNA (1 μg) was transfected into NOK cell lines, selected with G418 (Sigma) and pooled for establishment of a stable cell line (NOK-ELDR). The FOXM1 promoter containing 5′ regulatory sequence –955 to +45 bp relative to the FOXM1 transcriptional start site was cloned into pGL3 vector (21). The full-length CTCF expression construct (Fl-CTCF, 727 amino acids) was cloned into pCAGGS- 3 × FLAG vector (45). C′-terminal deleted construct of CTCF (Δ CTCF, 571 amino acids) was cloned into pCAGGS- 3 × FLAG plasmid using specific primers: FP: 5′ ACCGGCGGCTCTAGAGCCTCTGC 3′ and RP: 5′ CTTGCCATGGAATTCCGACGTGTAAA 3′ and XbaI and EcoRI restriction enzymes respectively. Cells were transfected with vector control or plasmids, mixed with Opti-MEM (Gibco) and Lipofectamine (Invitrogen) and incubated for 48 h.

The siRNAs for CTCF or ELDR were purchased from Dharmacon, Horizon Discovery. Cells were transfected with control oligo or siRNAs (100 nM), mixed with Opti-MEM (Gibco) and Lipofectamine RNAiMAX (Invitrogen), and incubated for 48 h. All the analyses were performed in triplicate.

### RNA isolation and qRT-PCR

Total RNA was isolated using TRIzol reagent (Invitrogen). cDNA was synthesized using a random hexamer with Superscript III reverse transcriptase (Thermo Fisher Scientific). The qRT-PCR was performed for quantitation of gene expression using specific primers (Table 1) by SYBR green-based detection system (Thermo Fisher Scientific) as per standard procedure. 18S rRNA was used as endogenous control. The relative gene expression was analyzed by using the 2^-ΔΔCT^ formula (ΔΔCT = ΔCT of the sample − ΔCT of the untreated control). Each sample was loaded in triplicate.

**Table 1:** Primer sequences

Gene	Sequence (5′- 3′)
ELDR	FP: ACTGAGATGAGACAGGTGGA RP: GAGCGATTTTTACACACCTT
AURKA	FP: AATAACACCCAAAAGAGCAA RP: AACTTTCCTTTACCCAGAGG
PLK1	FP: CGATACTACCTACGGCAAAT RP: CGGGAGCTATGTAATTAGGA
CDC25C	FP: GAAGAGGACAGGTCTCTGAA RP: CTCAGTCTTGTGGTCCTGAT
CDK1	FP: CTCCCAATAATGAAGTGTGG RP: GTTTGGCTGGATCATAGATT
CCNB1	FP: CCTGAGCCTGTTAAAGAAGA RP: TTCTGCATCCACATCATTTA
FOXM1	FP: ACCGCTACTTGACATTGGAC RP: GGAGTTCGGTTTTGATGGTC
CTCF	FP: AAACGTCACATTCGCTCTCA RP: GGGTAAACCGAGCATGACAA
NORAD	FP: AGCGAAGTCCCGAACGACGA RP: TGGGCATTTCCAACGGGCCAA
18s	FR: GTCATAAGCTTGCGTTGATT RP: TAGTCAAGTTCGACCGTCTT

### RNA sequence analysis and gene pathway and GSEA

For RNA seq analysis from total RNA, samples were prepared according to library kit manufacturer’s protocol, indexed, pooled, and sequenced on an Illumina HiSeq, and biological triplicates were used. The detailed method is discussed in the Supporting document. Pathway analysis was conducted using the Toppgene Server (https://toppgene.cchmc.org/) as described (46). Genes upregulated in NOK-ELDR compared to control NOK under the cut-offs of log_2_(fold change) > 3 and FDR <0.05 were identified with edgeR (47) and subsequently used for ToppGene pathway analysis. The returned pathways were ranked according to their FDR values (-log_10_FDR). GSEA (48) was performed as described using the “fgsea” R package (49). Fold changes and *p*-values were determined with edgeR and subsequently used to compute the rank of genes using the following formula: rank = -log_2_(p)∗sign(log_2_(fold change)). An enrichment score was calculated based on the cumulative rank of all genes of a given gene list in the PID (15). The *p*-values were calculated using the permutation test. The PID database was downloaded from the Molecular Signatures Database V5.2 (https://www.gsea-msigdb.org/gsea/msigdb/).

### Western blot analysis

Cell lysates were prepared using 2× SDS sample buffer, and Western blot analysis was performed using specific antibodies to AURKA (1: 1000, Cell Signaling Technology, CST), p-CDK1/p-Cdc2 (Tyr-15) (1: 1000, CST), total CDK1/Cdc2 (1: 1000, CST), CTCF (1: 1000, CST), and FOXM1 (1: 1000, CST). The HRP-conjugated anti mouse or anti-rabbit secondary antibodies (1:5000) were purchased from Bio-Rad. The blot was reprobed with Actin- HRP antibody (1:5000, Santa Cruz Biotechnology, SBT) to compare protein load in each lane. Densitometry analysis was performed using Image J software.

### Protein stability assay

NOK or NOK-ELDR was treated with 20 μg/ml cycloheximide (Sigma) to inhibit protein synthesis. Cycloheximide treated cells were harvested at different time points (0, 15, 30, 60, 90, 120, and 240 min) and processed for Western blot analysis for CTCF with specific antibody. Anti-actin antibody was used as internal control. In a separate experiment, NOK was treated with vehicle or 10 μM MG132, a proteasome inhibitor, followed by treatment with cycloheximide and cell lysate was collected at 0 and 5 h for CTCF expression analysis by Western blot.

### *In vitro* transcription and RNA pull-down assay

*In vitro* transcription and RNA pull-down assays were performed as described previously (9). Briefly, linearized ELDR-pcDNA3.1 plasmid DNA (500 ng) was *in vitro* transcribed using T7 (for sense strand) and SP6 (anti-sense strand) polymerase and NTP/Biotin- UTP mix at 37 °C for 3 h by AmpliScribe T7-Flash Biotin- RNA Transcription Kit (Lucigen-Epicentre). Newly synthesized biotin-labeled sense and anti-sense RNA was incubated with oral cancer cells Cal27 and JHU022 cells lysates, and RNA-pull down assay was performed by Pierce Magnetic RNA-Protein Pull-Down Kit (ThermoFisher Scientific). In brief, cell lysates were incubated with streptavidin magnetic beads-labeled ELDR sense and anti-sense RNA for 2 h at 4 °C. RNA beads–bound proteins were separated from whole lysates by a magnetic stand, washed, and eluted. The eluted supernatant against sense/antisense strand was examined by LC-MS and Western blot analysis. The LC-MS was done in the proteomics core facility, Washington University. Briefly, the mass spectrometer used for data acquisition was a Thermo Q-Exactive system. Peptides were separated on an EASYnLC system with a Thermo ES803 PepMap C18 column; data were acquired in data-dependent acquisition mode (top 10 m/z values for MS2 per cycle). Candidate proteins were defined as those having minimum five spectrum count and at least 2-fold enrichment compared to anti-sense ELDR RNA pull down. In another set of experiments, the eluted supernatant along with total lysates were subjected to Western blot analysis for detection of CTCF (1:1000, CST), Flag (1:1000, Sigma), and actin (1:5000, SBT).

### RNA immunoprecipitation assay

NOK-ELDR cells were lysed with IP lysis buffer (25 mM Tris, 150 mM NaCl, 1 mM EDTA, 1% Nonidet P-40, 5% glycerol, 1 × protease inhibitor cocktail, and 100 U/ml RNase inhibitor), and immunoprecipitation assay was done with the control IgG or anti-CTCF (CST) or PUM1 antibodies (CST) as described previously (9). Cell lysates were incubated with 5 μg of control IgG or anti-CTCF or anti-PUM1 antibodies for overnight at 4 °C followed by incubation with Protein G Sepharose beads (Amersham Bioscience) for 2 h at 4 °C. Immunoprecipitate complex was collected after centrifugation and washing with IP lysis buffer. Total RNA was isolated using TRIzol reagent, cDNA was synthesized, and relative mRNA expression of ELDR and NORAD (Table 1) was examined as described before (9).

### Subcellular fractionation

For subcellular fraction, NOK-ELDR cells were lysed in five pellet volumes of extraction buffer (10 mM Hepes, 60 mM KCl, 1 mM EDTA, 0.075% (v/v) NP-40, 1 mM DTT, and 1 mM PMSF, pH 7.6.) on ice for 3 min followed by centrifugation at 1500 rpms for 4 min. Cytoplasmic extract was separated, and nuclear pellet was washed gently with extraction buffer without NP-40. The nuclear pellet was then lysed by nuclear extraction buffer (20 mM Tris Cl, 420 mM NaCl, 1.5 mM MgCl_2_, 0.2 mM EDTA, 1 mM PMSF, and 25% (v/v) glycerol, pH 8.0) with additional 400 mM NaCl on ice for 10 min with periodic vortexing. Finally, both cytosolic and nuclear fractions were centrifuged at maximum speed for 10 min to pellet any nuclei. The clear supernatant was divided into two, one for immunoprecipitation assay with CTCF antibody followed by ELDR mRNA expression analysis from the precipitates as described above. Another half was subjected to Western blot using 2× SDS sample buffer as described before for detection of GAPDH (1:5000, CST) (cytoplasmic marker) and lamin A/C (1:500, SBT) (nuclear marker).

### Immunofluorescence analysis

NOK or NOK-ELDR were fixed with ice-cold methanol, and immunofluorescence analysis was performed as described previously (9). Cells were permeabilized with 0.1% Triton x100 for 10 min followed by incubation with 5% bovine serum albumin for 2 h at room temperature for blocking. Then, the cells were incubated with respective primary antibodies [phospho histone H3 (Ser10) (1:100, CST), CTCF (CST, 1: 100), and Tubulin (1:100, SBT)], followed by incubation with anti-rabbit immunoglobulin conjugated to Alexa Fluor 594 and/or anti-mouse immunoglobulin conjugated to Alexa Fluor 488 (Molecular Probes). Cells were counter stained with 1 μg/ml 4′, 6-diamidino-2-phenylindole (DAPI) for nuclear staining and observed under Olympus FV1000 confocal microscope or fluorescence microscope (BZ-X800, Keyence). For negative control, same procedure was followed without addition of primary antibody.

### Flow cytometry analysis

Synchronized NOK and NOK-ELDR cells were incubated with propidium iodide solution (50 μg/ml propidium iodide, 0.1% Triton X-100 and 10 μg/ml RNase A) for 1 h at room temperature, and flow cytometry analysis was performed on a FACScan flow cytometer (BD PharMingen) as described previously (9). Data were analyzed using the CellQuest and ModFit software.

### Luciferase assays

For luciferase assay, NOK, NOK-ELDR, and 293T cells were transfected with the FOXM1 luciferase reporter plasmid along with/without ELDR, CTCF, siCTCF, or respective controls. Cell extract was prepared 48 h after transfection, and relative luciferase activity was determined as described by us previously (50).

### Statistical analysis

The results are presented as means ± standard deviations. Data were analyzed by Student's *t* test [two-tailed *t* test]. *p*-value of <0.05 was considered statistically significant. In each experiment, we used biological and technical triplicates, and representative data are shown.

## Data availability

Data generated in this study are included in the article or supporting information. Geo accession number: GSE198818

## Supporting information

This article contains supporting information (47, 51, 52, 53, 54, 55, 56, 57, 58, 59, 60, 61, 62).

## Conflict of interest

The authors declare that they have no conflicts of interest with the contents of this article.

## References

[1] Consortium, E. P. (2012). An integrated encyclopedia of DNA elements in the human genome. Nature.

[2] Gloss B.S., Dinger M.E. (2018). Realizing the significance of noncoding functionality in clinical genomics. Exp. Mol. Med..

[3] Statello L., Guo C.J., Chen L.L., Huarte M. (2020). Gene regulation by long non-coding RNAs and its biological functions. Nat. Rev. Mol. Cell Biol..

[4] Irimie A.I., Braicu C., Sonea L., Zimta A.A., Cojocneanu-Petric R., Tonchev K., Mehterov N., Diudea D., Buduru S., Berindan-Neagoe I. (2017). A Looking-Glass of Non-coding RNAs in oral cancer. Int. J. Mol. Sci..

[5] Shih J.W., Chiang W.F., Wu A.T.H., Wu M.H., Wang L.Y., Yu Y.L., Hung Y.W., Wang W.C., Chu C.Y., Hung C.L., Changou C.A., Yen Y., Kung H.J. (2017). Long noncoding RNA LncHIFCAR/MIR31HG is a HIF-1alpha co-activator driving oral cancer progression. Nat. Commun..

[6] Zhang L., Meng X., Zhu X.W., Yang D.C., Chen R., Jiang Y., Xu T. (2019). Long non-coding RNAs in oral squamous cell carcinoma: Biologic function, mechanisms and clinical implications. Mol. Cancer.

[7] Nishiyama K., Maruyama R., Niinuma T., Kai M., Kitajima H., Toyota M., Hatanaka Y., Igarashi T., Kobayashi J.I., Ogi K., Dehari H., Miyazaki A., Yorozu A., Yamamoto E., Idogawa M. (2018). Screening for long noncoding RNAs associated with oral squamous cell carcinoma reveals the potentially oncogenic actions of DLEU1. Cell Death Dis..

[8] Aznaourova M., Schmerer N., Schmeck B., Schulte L.N. (2020). Disease-causing mutations and rearrangements in long non-coding RNA gene loci. Front. Genet..

[9] Sur S., Nakanishi H., Steele R., Zhang D., Varvares M.A., Ray R.B. (2020). Long non-coding RNA ELDR enhances oral cancer growth by promoting ILF3-cyclin E1 signaling. EMBO Rep..

[10] Sauvageau M., Goff L.A., Lodato S., Bonev B., Groff A.F., Gerhardinger C., Sanchez-Gomez D.B., Hacisuleyman E., Li E., Spence M., Liapis S.C., Mallard W., Morse M., Swerdel M.R., D'Ecclessis M.F. (2013). Multiple knockout mouse models reveal lincRNAs are required for life and brain development. Elife.

[11] Goff L.A., Groff A.F., Sauvageau M., Trayes-Gibson Z., Sanchez-Gomez D.B., Morse M., Martin R.D., Elcavage L.E., Liapis S.C., Gonzalez-Celeiro M., Plana O., Li E., Gerhardinger C., Tomassy G.S., Arlotta P. (2015). Spatiotemporal expression and transcriptional perturbations by long noncoding RNAs in the mouse brain. Proc. Natl. Acad. Sci. U. S. A..

[12] Carelli S., Giallongo T., Rey F., Latorre E., Bordoni M., Mazzucchelli S., Gorio M.C., Pansarasa O., Provenzani A., Cereda C., Di Giulio A.M. (2019). HuR interacts with lincBRN1a and lincBRN1b during neuronal stem cells differentiation. RNA Biol..

[13] Nikonova A.S., Astsaturov I., Serebriiskii I.G., Dunbrack R.L., Golemis E.A. (2013). Aurora A kinase (AURKA) in normal and pathological cell division. Cell Mol. Life Sci..

[14] Dawei H., Honggang D., Qian W. (2018). AURKA contributes to the progression of oral squamous cell carcinoma (OSCC) through modulating epithelial-to-mesenchymal transition (EMT) and apoptosis *via* the regulation of ROS. Biochem. Biophys. Res. Commun..

[15] Schaefer C.F., Anthony K., Krupa S., Buchoff J., Day M., Hannay T., Buetow K.H. (2009). Pid: The pathway interaction database. Nucleic Acids Res..

[16] Puig-Butille J.A., Vinyals A., Ferreres J.R., Aguilera P., Cabre E., Tell-Marti G., Marcoval J., Mateo F., Palomero L., Badenas C., Piulats J.M., Malvehy J., Pujana M.A., Puig S., Fabra A. (2017). AURKA overexpression is driven by FOXM1 and MAPK/ERK activation in melanoma cells harboring BRAF or NRAS mutations: Impact on melanoma prognosis and therapy. J. Invest. Dermatol..

[17] Down C.F., Millour J., Lam E.W., Watson R.J. (2012). Binding of FoxM1 to G2/M gene promoters is dependent upon B-Myb. Biochim. Biophys. Acta.

[18] Jaiswal N., Chakraborty S., Nag A. (2014). Biology OF FOXM1 and its emerging role IN cancer therapy. J. Proteins Proteomics.

[19] Liao G.B., Li X.Z., Zeng S., Liu C., Yang S.M., Yang L., Hu C.J., Bai J.Y. (2018). Regulation of the master regulator FOXM1 in cancer. Cell Commun. Signal..

[20] Waseem A., Ali M., Odell E.W., Fortune F., Teh M.T. (2010). Downstream targets of FOXM1: CEP55 and HELLS are cancer progression markers of head and neck squamous cell carcinoma. Oral Oncol..

[21] Zhang B., Zhang Y., Zou X., Chan A.W., Zhang R., Lee T.K., Liu H., Lau E.Y., Ho N.P., Lai P.B., Cheung Y.S., To K.F., Wong H.K., Choy K.W., Keng V.W. (2017). The CCCTC-binding factor (CTCF)-forkhead box protein M1 axis regulates tumour growth and metastasis in hepatocellular carcinoma. J. Pathol..

[22] Weth O., Renkawitz R. (2011). CTCF function is modulated by neighboring DNA binding factors. Biochem. Cell Biol..

[23] Saldana-Meyer R., Rodriguez-Hernaez J., Escobar T., Nishana M., Jacome-Lopez K., Nora E.P., Bruneau B.G., Tsirigos A., Furlan-Magaril M., Skok J., Reinberg D. (2019). RNA interactions are essential for CTCF-mediated genome organization. Mol. Cell.

[24] Tichon A., Gil N., Lubelsky Y., Havkin Solomon T., Lemze D., Itzkovitz S., Stern-Ginossar N., Ulitsky I. (2016). A conserved abundant cytoplasmic long noncoding RNA modulates repression by Pumilio proteins in human cells. Nat. Commun..

[25] Piboonniyom S.O., Duensing S., Swilling N.W., Hasskarl J., Hinds P.W., Munger K. (2003). Abrogation of the retinoblastoma tumor suppressor checkpoint during keratinocyte immortalization is not sufficient for induction of centrosome-mediated genomic instability. Cancer Res..

[26] Bhan S., Chuang A., Negi S.S., Glazer C.A., Califano J.A. (2012). MAGEA4 induces growth in normal oral keratinocytes by inhibiting growth arrest and apoptosis. Oncol. Rep..

[27] Sanz-Gomez N., de Pedro I., Ortigosa B., Santamaria D., Malumbres M., de Carcer G., Gandarillas A. (2020). Squamous differentiation requires G2/mitosis slippage to avoid apoptosis. Cell Death Differ..

[28] Krayzler E., Nagler R.M. (2015). Cigarette smoke-induced effects on the cell cycle in oral cancer cells: Reduction of G2/M fraction. Cancer Genomics Proteomics.

[29] Tsou H.H., Tsai H.C., Chu C.T., Cheng H.W., Liu C.J., Lee C.H., Liu T.Y., Wang H.T. (2021). Cigarette smoke containing acrolein upregulates EGFR signaling contributing to oral tumorigenesis *in vitro* and *in vivo*. Cancers (Basel).

[30] Hanahan D., Weinberg R.A. (2011). Hallmarks of cancer: The next generation. Cell.

[31] Du R., Huang C., Liu K., Li X., Dong Z. (2021). Targeting AURKA in cancer: Molecular mechanisms and opportunities for cancer therapy. Mol. Cancer.

[32] Liu Q., Ruderman J.V. (2006). Aurora A, mitotic entry, and spindle bipolarity. Proc. Natl. Acad. Sci. U. S. A..

[33] Katayama H., Brinkley W.R., Sen S. (2003). The Aurora kinases: Role in cell transformation and tumorigenesis. Cancer Metastasis Rev..

[34] Gartel A.L. (2017). FOXM1 in cancer: Interactions and vulnerabilities. Cancer Res..

[35] Gentles A.J., Newman A.M., Liu C.L., Bratman S.V., Feng W., Kim D., Nair V.S., Xu Y., Khuong A., Hoang C.D., Diehn M., West R.B., Plevritis S.K., Alizadeh A.A. (2015). The prognostic landscape of genes and infiltrating immune cells across human cancers. Nat. Med..

[36] Wierstra I. (2013). FOXM1 (forkhead box M1) in tumorigenesis: Overexpression in human cancer, implication in tumorigenesis, oncogenic functions, tumor-suppressive properties, and target of anticancer therapy. Adv. Cancer Res..

[37] Teh M.T., Gemenetzidis E., Patel D., Tariq R., Nadir A., Bahta A.W., Waseem A., Hutchison I.L. (2012). FOXM1 induces a global methylation signature that mimics the cancer epigenome in head and neck squamous cell carcinoma. PLoS One.

[38] Chow A. (2010). Cell cycle control by oncogenes and tumor suppressors: Driving the transformation of normal cells into cancerous cells. Nat. Educ..

[39] Gavilan E., Giraldez S., Sanchez-Aguayo I., Romero F., Ruano D., Daza P. (2015). Breast cancer cell line MCF7 escapes from G1/S arrest induced by proteasome inhibition through a GSK-3beta dependent mechanism. Sci. Rep..

[40] Ren G., Zhao K. (2019). CTCF and cellular heterogeneity. Cell Biosci..

[41] Bailey C.G., Metierre C., Feng Y., Baidya K., Filippova G.N., Loukinov D.I., Lobanenkov V.V., Semaan C., Rasko J.E. (2018). CTCF expression is essential for somatic cell viability and protection against cancer. Int. J. Mol. Sci..

[42] Aitken S.J., Ibarra-Soria X., Kentepozidou E., Flicek P., Feig C., Marioni J.C., Odom D.T. (2018). CTCF maintains regulatory homeostasis of cancer pathways. Genome Biol..

[43] Lai Q., Li Q., He C., Fang Y., Lin S., Cai J., Ding J., Zhong Q., Zhang Y., Wu C., Wang X., He J., Liu Y., Yan Q., Li A. (2020). CTCF promotes colorectal cancer cell proliferation and chemotherapy resistance to 5-FU *via* the P53-Hedgehog axis. Aging (Albany NY).

[44] Qi C.F., Kim Y.S., Xiang S., Abdullaev Z., Torrey T.A., Janz S., Kovalchuk A.L., Sun J., Chen D., Cho W.C., Gu W., Morse H.C. (2012). Characterization of ARF-BP1/HUWE1 interactions with CTCF, MYC, ARF and p53 in MYC-driven B cell neoplasms. Int. J. Mol. Sci..

[45] Sekiya T., Murano K., Kato K., Kawaguchi A., Nagata K. (2017). Mitotic phosphorylation of CCCTC-binding factor (CTCF) reduces its DNA binding activity. FEBS Open Bio..

[46] Steinauer N., Guo C., Huang C., Wong M., Tu Y., Freter C.E., Zhang J. (2019). Myeloid translocation gene CBFA2T3 directs a relapse gene program and determines patient-specific outcomes in AML. Blood Adv..

[47] Robinson M.D., McCarthy D.J., Smyth G.K. (2010). edgeR: a Bioconductor package for differential expression analysis of digital gene expression data. Bioinformatics.

[48] Subramanian A., Tamayo P., Mootha V.K., Mukherjee S., Ebert B.L., Gillette M.A., Paulovich A., Pomeroy S.L., Golub T.R., Lander E.S., Mesirov J.P. (2005). Gene set enrichment analysis: A knowledge-based approach for interpreting genome-wide expression profiles. Proc. Natl. Acad. Sci. U. S. A..

[49] Sergushichev A.A. (2016). Algorithm for cumulative calculation of gene set enrichment statistic. Sci. Tech. J. Inf. Tech. Mech. Optics.

[50] Ray R.B., Steele R., Meyer K., Ray R. (1998). Hepatitis C virus core protein represses p21WAF1/Cip1/Sid1 promoter activity. Gene.

[51] Dobin A., Davis C.A., Schlesigner F., Drenkow J., Zaleski C., Jha S., Batut P., Chaisson M., Gingeras T.R. (2013). STAR: Ultrafast universal RNA-seq aligner. Bioinformatics.

[52] Liao Y., Smyth G.K., Shi W. (2014). featureCounts: An efficient general purpose program for assigning sequence reads to genomic features. Bioinformatics.

[53] Patro R., Duggal G., Love M.I., Irizarry R.A., Kingsford C. (2017). Salmon provides fast and bias-aware quantification of transcript expression. Nat. Methods.

[54] Wang L., Wang S., Li W. (2012). RSeQC: Quality control of RNA-seq experiments. Bioinformatics.

[55] Ritchie M.E., Phipson B., Wu D., Hu Y., Law C.W., Shi W., Smyth G.K. (2015). limma powers differential expression analyses for RNA-sequencing and microarray studies. Nucleic Acids Res..

[56] Liu R., Holik A.Z., Su S., Jansz N., Chen K., Leong H.S., Blewitt M.E., Asselin-Labat M.-L., Smyth G.K., Ritchie M.E. (2015). Why weight? Modelling sample and observational level variability improves power in RNA-seq analyses. Nucleic Acids Res..

[57] Luo W., Friedman M., Shedden K., Hankenson K., Woolf P. (2009). GAGE: Generally applicable gene set enrichment for pathway analysis. BMC Bioinformatics.

[58] Zhao S., Guo Y., Sheng Q., Shyr Y. (2014). Advanced heat map and clustering analysis using heatmap3. Biomed Res. Int..

[59] Luo W., Brouwer C. (2013). Pathview: An R/Bioconductor package for pathway-based data integration and visualization. Bioinformatics.

[60] Love M.I., Huber W., Anders S. (2014). Moderated estimation of fold change and dispersion for RNA-seq data with DESeq2. Genome Biol..

[61] Langfelder P., Horvath S. (2018). WGCNA: An R package for weighted correlation network analysis. BMC Bioinformatics.

[62] Yu G., Wang L., Han Y., He Q. (2012). clusterProfiler: An R package for comparing biological themes among gene clusters. OMICS.

