# Supplementary material for: Long noncoding RNA ELDR promotes cell cycle progression in normal oral keratinocytes through induction of a CTCF-FOXM1-AURKA signaling axis
Source: J Biol Chem. 2022 Apr 1;298(5):101895. doi: 10.1016/j.jbc.2022.101895 (PMC9079251; doi:10.1016/j.jbc.2022.101895)
Supplement: Supplemental Figures S1–S7 [file mmc1.pdf]

Supporting Document

Long non-coding RNA ELDR promotes cell cycle progression in normal oral keratinocytes through induction of a CTCF-FOXM1-AURKA signaling axis

Subhayan Sur[1], Robert Steele[1], Ben C. B. Ko[2], Jinsong Zhang[3], and Ratna B. Ray[1, *]
Departments of Pathology[1], and Pharmacology and Physiology[3], Saint Louis University, Missouri, USA, and Department of Applied Biology and Chemical Technology[2], The Hong Kong Polytechnic University, Hong Kong, SAR, PR China

RNA Sequencing and Analysis

Samples were prepared according to library kit manufacturer's protocol, indexed, pooled, and sequenced on an Illumina HiSeq. Basecalls and demultiplexing were performed with Illumina's bcl2fastq software and a custom python demultiplexing program with a maximum of one mismatch in the indexing read. RNA-seq reads were then aligned to the Ensembl release 76 primary assembly with STAR version 2.5.1a (1). Gene counts were derived from the number of uniquely aligned unambiguous reads by Subread:featureCount version 1.4.6-p5 (2). Isoform expression of known Ensembl transcripts were estimated with Salmon version 0.8.2 (3). Sequencing performance was assessed for the total number of aligned reads, total number of uniquely aligned reads, and features detected. The ribosomal fraction, known junction saturation, and read distribution over known gene models were quantified with RSeQC version 2.6.2 (4).

All gene counts were then imported into the R/Bioconductor package EdgeR (5) and TMM normalization size factors were calculated to adjust for samples for differences in library size. Ribosomal genes and genes not expressed in the smallest group size minus one samples greater than one count-per-million were excluded from further analysis. The TMM size factors and the matrix of counts were then imported into the R/Bioconductor package Limma (6). Weighted likelihoods based on the observed mean-variance relationship of every gene and sample were then calculated for all samples with the voomWithQualityWeights (7). The performance of all genes was assessed with plots of the residual standard deviation of every gene to their average log-count with a robustly fitted trend line of the residuals. Differential expression analysis was then performed to analyze for differences between conditions and the results were filtered for only those genes with Benjamini-Hochberg false-discovery rate adjusted p-values less than or equal to 0.05.

For each contrast extracted with Limma, global perturbations in known Gene Ontology (GO) terms, MSigDb, and KEGG pathways were detected using the R/Bioconductor package GAGE (8) to test for changes in expression of the reported log 2 fold-changes reported by Limma in each term versus the background log 2 fold-changes of all genes found outside the respective term. The R/Bioconductor package heatmap3 (9) was used to display heatmaps across groups of samples for each GO or MSigDb term with a Benjamini-Hochberg false-discovery rate adjusted p-value less than or equal to 0.05. Perturbed KEGG pathways where the observed log 2 fold-changes of genes within the term were significantly perturbed in a single-direction versus background or in any direction compared to other genes within a given term with p-values less than or equal to 0.05 were rendered as nnotated KEGG graphs with the R/Bioconductor package Pathview (10).

To find the most critical genes, the raw counts were variance stabilized with the R/Bioconductor package DESeq2 (11) and was then analyzed via weighted gene correlation network analysis with the R/Bioconductor package WGCNA (12). Briefly, all genes were correlated across each other by Pearson correlations and clustered by expression similarity into unsigned modules using a power threshold empirically determined from the data. An eigengene was then created for each de novo cluster and its expression profile was then correlated across all coefficients of the model matrix. Because these clusters of genes were created by expression profile rather than known functional similarity, the clustered modules were given the names of random colors where grey is the only module that has any pre-existing definition of containing genes that do not cluster well

with others. These de-novo clustered genes were then tested for functional enrichment of known GO terms with hypergeometric tests available in the R/Bioconductor package clusterProfiler (13). Significant terms with Benjamini-Hochberg adjusted p-values less than 0.05 were then collapsed by similarity into clusterProfiler category network plots to display the most significant terms for each module of hub genes in order to interpolate the function of each significant module. The information for all clustered genes for each module were then combined with their respective statistical significance results from Limma to determine whether or not those features were also found to be significantly differentially expressed.

References

1. Dobin, A., Davis, CA, Schlesigner, F., Drenkow, J., Zaleski, C., Jha, S., Batut, P., Chaisson, M., Gingeras, TR. STAR: ultrafast universal RNA-seq aligner. Bioinformatics 29, 15-21 (2013).

2. Liao, Y, Smyth, GK and Shi, W. featureCounts: an efficient general purpose program for assigning sequence reads to genomic features. Bioinformatics, 30:923-30 (2014).

3. Patro, R., Duggal, G., Love, M. I., Irizarry, R. A., & Kingsford, C. Salmon provides fast and bias-aware quantification of transcript expression. Nature Methods 14 (2017).

4. Wang, L., Wang, S., & Li, W. RSeQC: quality control of RNA-seq experiments. Bioinformatics 28, 2184–2185 (2012).

5. Robinson MD, McCarthy DJ, Smyth GK. edgeR: a Bioconductor package for differential expression analysis of digital gene expression data. Bioinformatics, 26(1), 139-140 (2010).

6. Ritchie, ME, Phipson, B, Wu, D, Hu, Y, Law, CW, Shi, W, and Smyth, GK. limma powers differential expression analyses for RNA-sequencing and microarray studies. Nucleic Acids Research 43 (2015).

7. Liu, R, Holik, AZ, Su, S, Jansz, N, Chen, K, Leong, HS, Blewitt, ME, Asselin-Labat, M-L, Smyth, GK, Ritchie, ME. Why weight? Modelling sample and observational level variability improves power in RNA-seq analyses. Nucleic Acids Research 43 (2015).

8. Luo, Weijun, Friedman, Michael, Shedden, Kerby, Hankenson, Kurt, Woolf, Peter. GAGE: generally applicable gene set enrichment for pathway analysis. BMC Bioinformatics, 10, 161 (2009).

9. Zhao, S., Guo Y., Sheng, Q., Shyr, Y. Advanced Heat Map and Clustering Analysis Using Heatmap3. Biomed Research International, 2014, 6 pages (2014).

10. Luo, Weijun, Brouwer, Cory. Pathview: an R/Bioconductor package for pathway-based data integration and visualization. Bioinformatics, 29(14), 1830-1831 (2013).

11. Love MI, Huber W, Anders S. Moderated estimation of fold change and dispersion for RNA-seq data with DESeq2. Genome Biology, 15, 550 (2014).

12. Langfelder, P and Horvath, S. WGCNA: an R package for weighted correlation network analysis. BMC Bioinformatics 9 (2018).

13. Yu G, Wang L, Han Y, He Q. clusterProfiler: an R package for comparing biological themes among gene clusters. OMICS: A Journal of Integrative Biology, 16(5), 284-287 (2012).

Supporting Figure Legends

Supporting Figure S1: Control or ELDR overexpressed NOK lysates were subjected to Western blot analysis for phospho histone H3 (pHH3) using specific antibody (1: 1000, Cell Signaling Technology). The membrane was reprobed with Actin as an internal control. Experiments were repeated two times.

Supporting Figure S2: Volcano plot from the mass spectrometry data demonstrating magnitude and significance of the proteins interacted with sense strand of ELDR compared with the antisense strand of ELDR in JHU022 and Cal27 cells. The red dot indicates CTCF protein. The x axis is the Log_2-fold change value and y axis is (−) Log_{10} statistical P value. The horizontal dashed line shows $P = 0.05$ ($-log_{10}[0.05] = 1.3$), and the vertical dashed line shows the fold change at 2 ($log_2[2] = 1$). The absolute 2-fold change and P value 0.05 are used as the threshold cutoff.

Supporting Figure S3. A: Schematic diagram of human full length CTCF construct (Fl-CTCF) and deleted construct (Δ CTCF). Black box indicates potential binding site of ELDR (626-677 amino acids). **B:** Lysates from NOK-ELDR cells transfected with either Flag-Fl-CTCF or Flag- Δ CTCF were incubated with biotinylated ELDR sense RNA, pulled down and subjected to Western blot analysis for Flag using specific antibody (1:1000, Sigma). The membrane was reporbed with Actin as internal control in total lysates. Experiments were repeated two times.

Supporting Figure S4. A: The expression of CTCF, AURKA and FOXM1 were shown among a total 15219 genes in the Volcano plot generated from the RNA seq data of NOK-ELDR vs. NOK. The x axis is the Log_2-fold change value and y axis is (−) Log_{10} false discovery rate (FDR). **B:** Relative mRNA expression of CTCF was analyzed by qRT-PCR in NOK and ELDR over expressed NOK. 18S rRNA was used as an internal control. Experiments were repeated two times with technical triplicates. Small bar indicates standard error.

Supporting Figure S5. A: Cytoplasmic and nuclear fractions of NOK-ELDR were immunoprecipitated against CTCF antibody and RNA was isolated from the precipitates. Relative expressions of ELDR were analyzed by qRT-PCR. Experiments were repeated two times with technical triplicates. Small bar indicates standard error (* $p<0.05$). **B:** Cytoplasmic and nuclear extract were subjected to Western blot analysis for GAPDH (1: 5000, Cell Signaling Technology). The membrane was reprobed for Lamin a/c (1:500, Santa Cruz Biotechnology).

Supporting Figure S6: NOK-ELDR cells were transfected with either control or two different siRNAs to CTCF and after 48h cells were harvested and relative mRNA expression of CTCF was analyzed by qRT-PCR. 18S was used as internal control. Experiments were repeated two times with technical triplicates. Small bar indicates standard error (* $p<0.05$; ** $p<0.01$).

Supporting Figure S7: NOKs were transfected with either control or CTCF plasmid and after 48h cells were harvested and relative mRNA expression of **(A)** CTCF, and **(B)** FOXM1 and AURKA were analyzed by qRT-PCR. 18S was used as internal control. Experiments were repeated three times with technical triplicates. Small bar indicates standard error (* $p<0.05$; ** $p<0.01$; ***$p<0.001$).

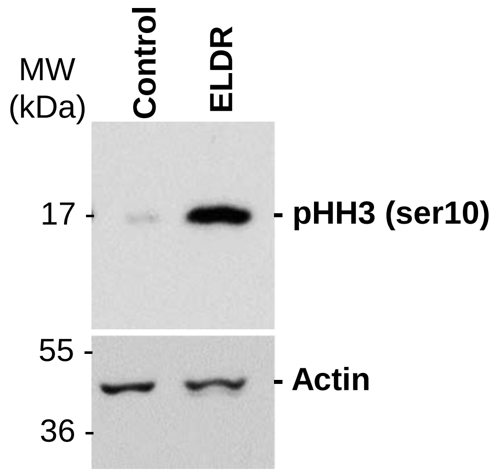

Figure S1

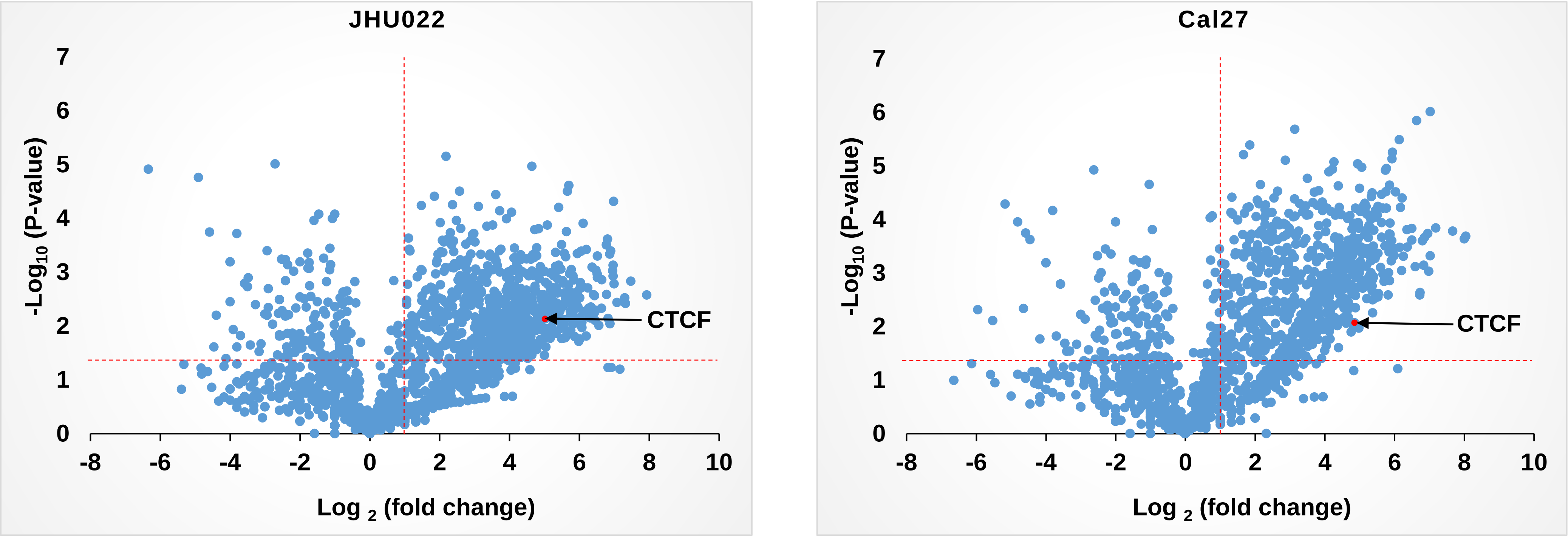
JHU022
Cal27
7
6
5
4
3
2
1
0
-Log10 (P-value)
-8 -6 -4 -2 0 2 4 6 8 10
Log 2 (fold change)
CTCF

Figure S2

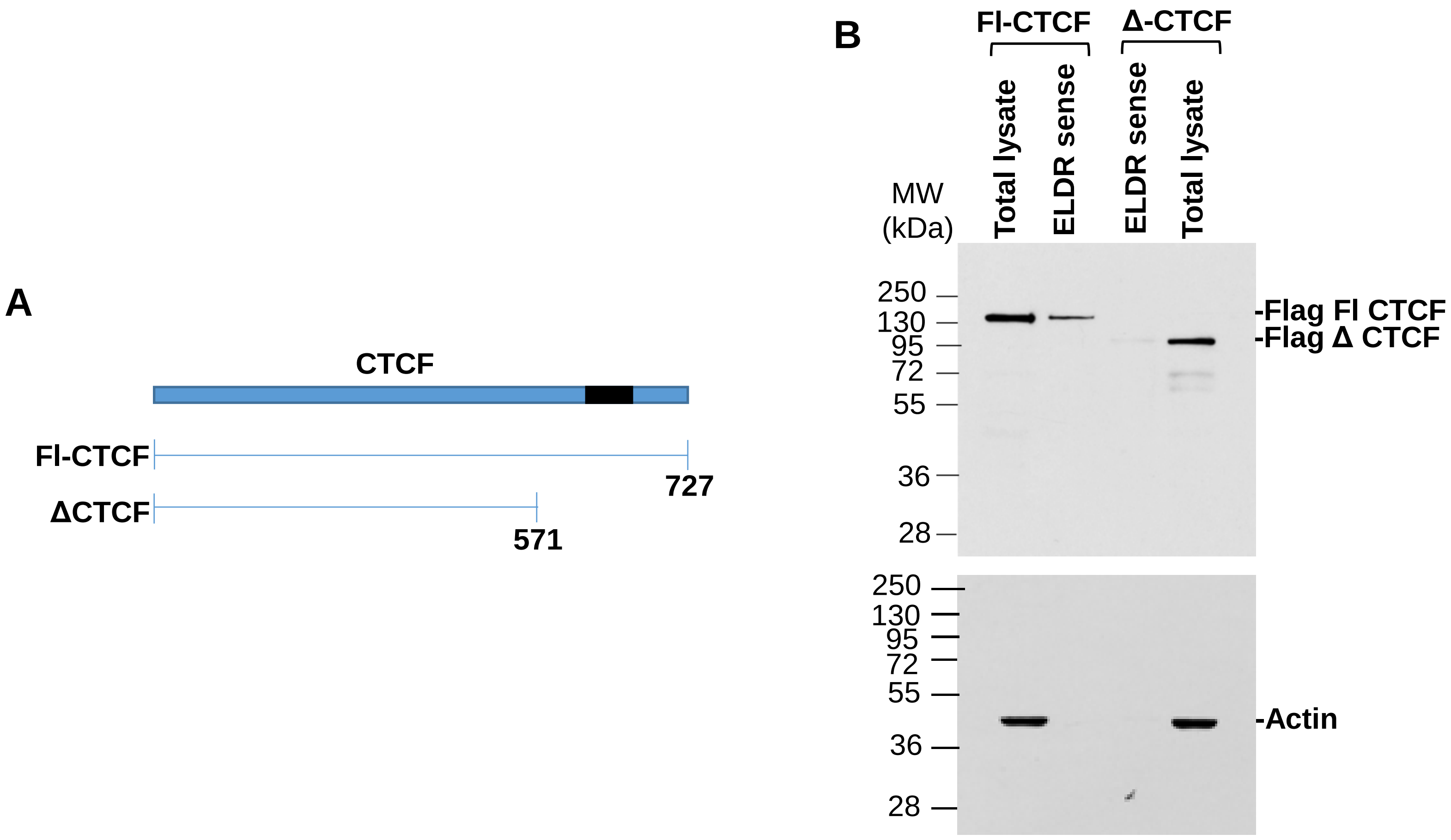

A
CTCF
Fl-CTCF
727
ΔCTCF
571
B
Fl-CTCF
Δ-CTCF
Total lysate
ELDR sense
ELDR sense
Total lysate
MW
(kDa)
250
130
95
72
55
36
28
-Flag Fl CTCF
-Flag Δ CTCF
250
130
95
72
55
36
28
-Actin

Figure S3

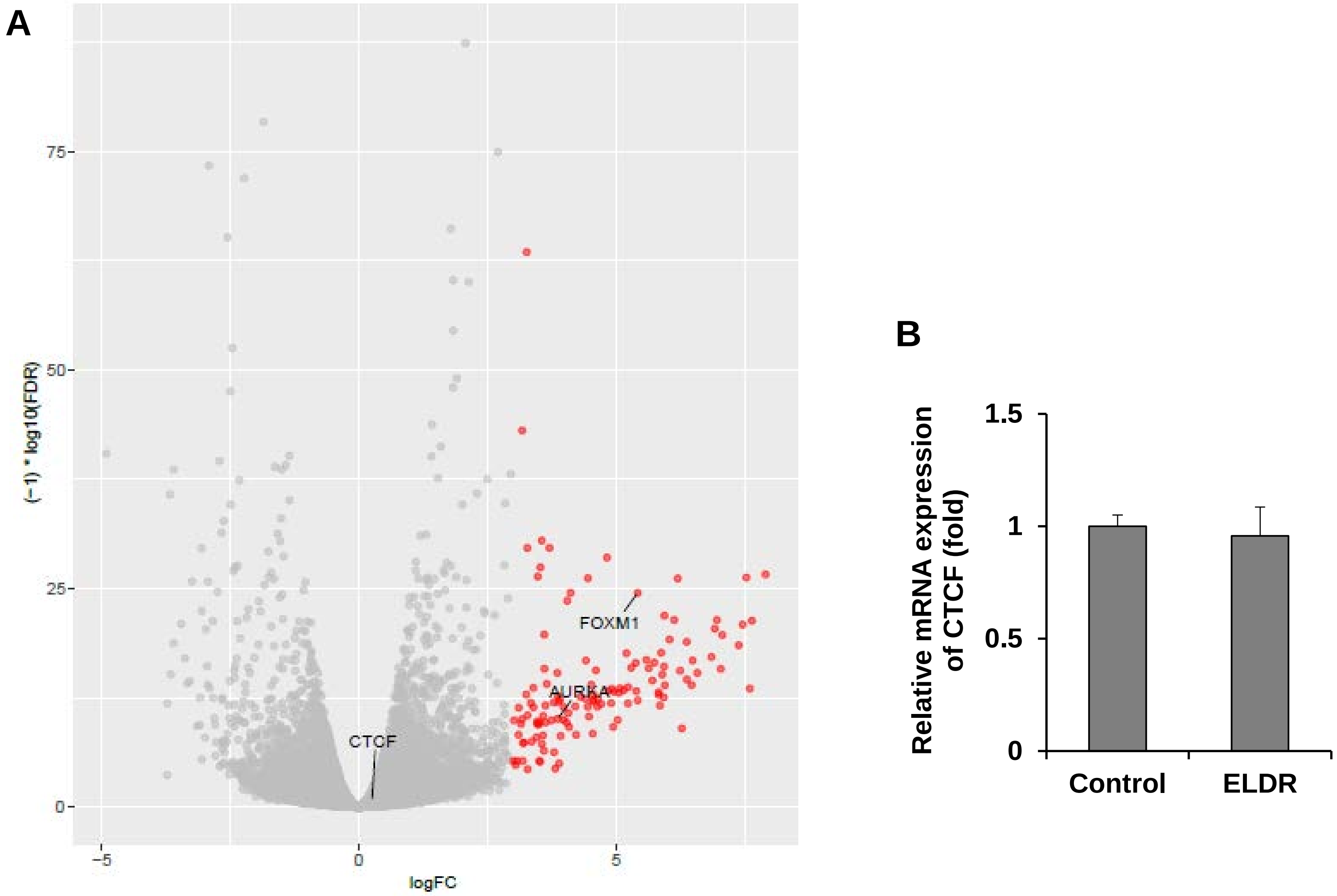
A
75
50
25
0
(−1) * log10(FDR)
−5
0
5
logFC
FOXM1
AURKA
CTCF
B
Relative mRNA expression of CTCF (fold)
1.5
1
0.5
0
Control
ELDR

Figure S4

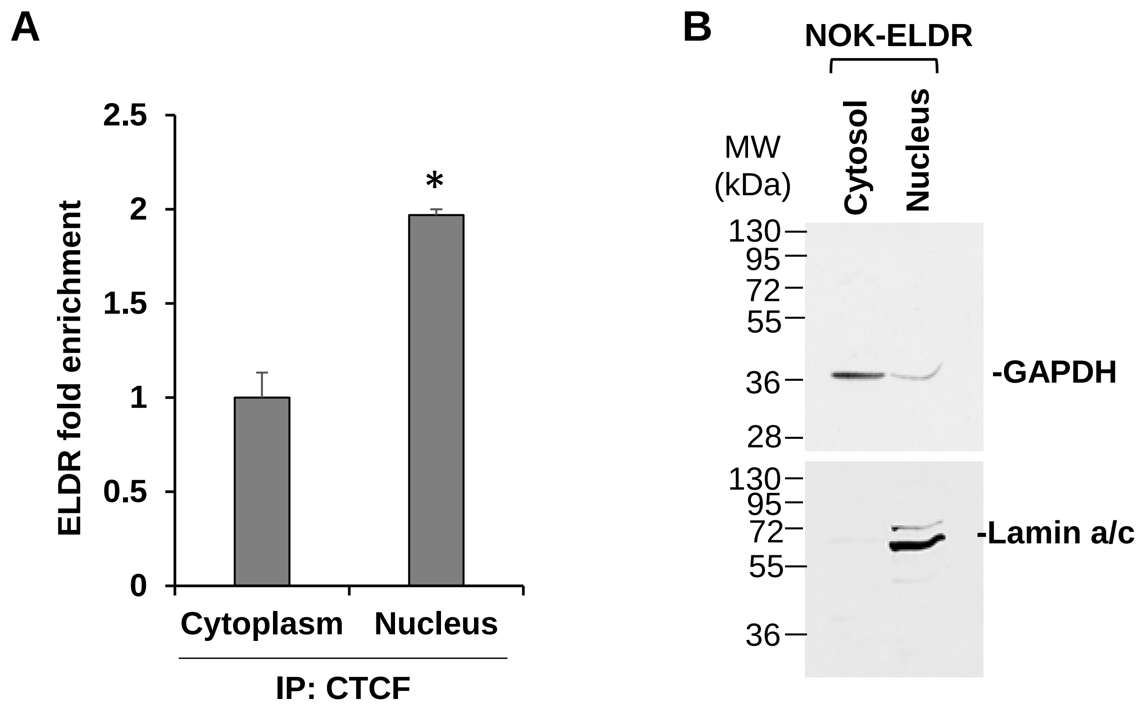
A
2.5
2
1.5
1
0.5
0
ELDR fold enrichment
*
Cytoplasm
Nucleus
IP: CTCF
B
NOK-ELDR
MW
(kDa)
Cytosol
Nucleus
130
95
72
55
36
28
-GAPDH
130
95
72
55
36
-Lamin a/c

Figure S5

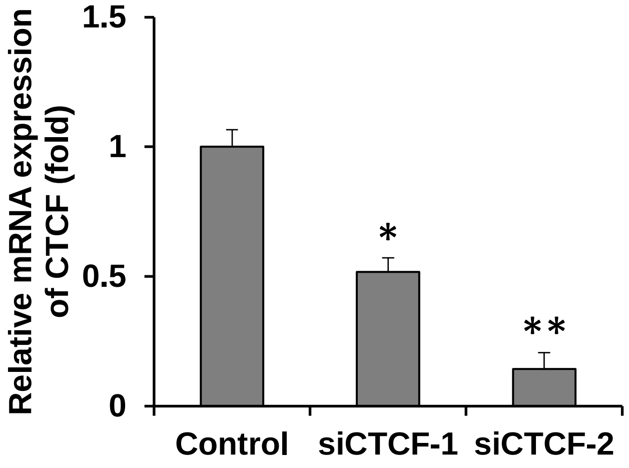

Relative mRNA expression of CTCF (fold)
1.5
1
0.5
0
*
**
Control
siCTCF-1
siCTCF-2

Figure S6

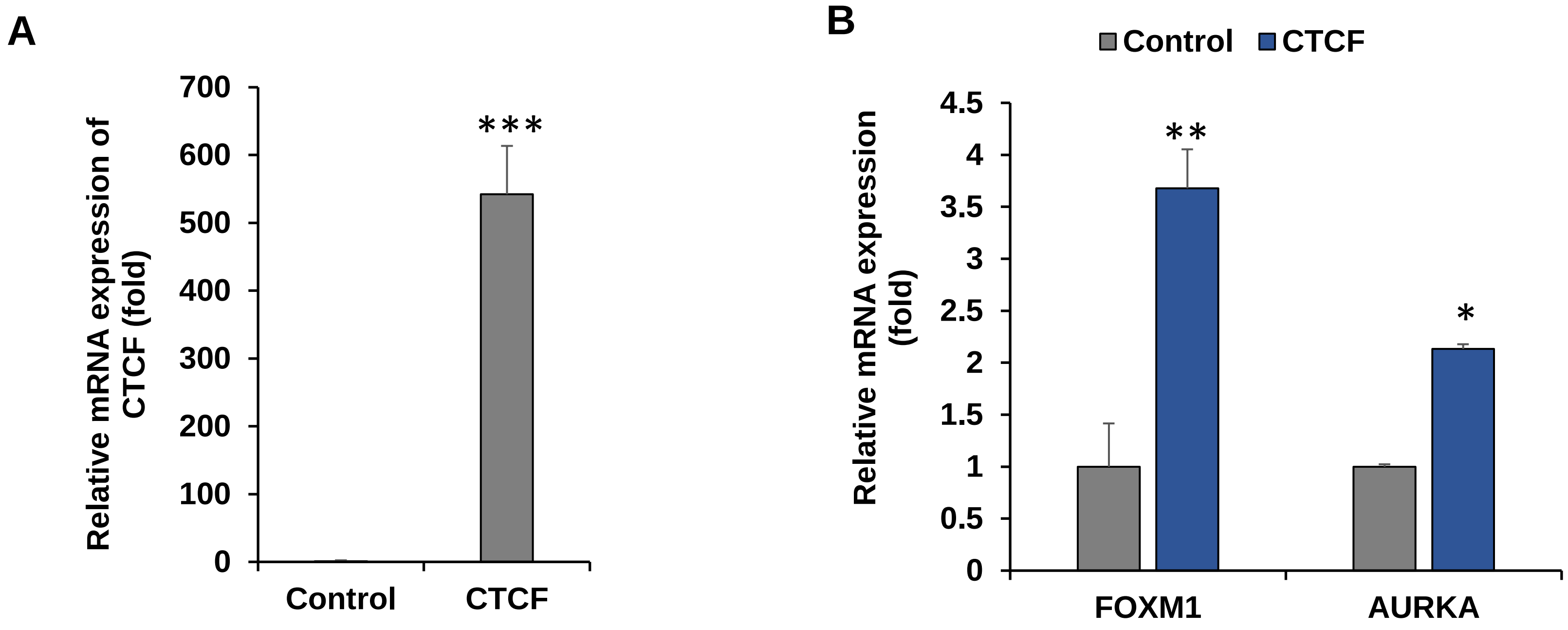
A
Relative mRNA expression of CTCF (fold)
700
600
500
400
300
200
100
0
***
Control
CTCF
B
Control
CTCF
Relative mRNA expression (fold)
4.5
4
3.5
3
2.5
2
1.5
1
0.5
0
**
*
FOXM1
AURKA

Figure S7
